# Phytochemical Profiling and Bioactivity Evaluation of *Ganoderma lucidum* (Reishi Mushroom) Fractions: In Vitro Antioxidant, Antimicrobial, and Antidiabetic Activities

**DOI:** 10.3390/metabo16040225

**Published:** 2026-03-30

**Authors:** Neelum Shehzadi, Sarmir Khan, Leonardo Degennaro, Gabriele D’Arienzo, Noshaba Mehmood, Aqsa Chaudhary, Muhammad Afzal, Maria Pia Argentieri

**Affiliations:** 1 Department of Basic and Applied Chemistry, Faculty of Science and Technology, University of Central Punjab, Lahore 54000, Pakistan; l1f21msbc0025@ucp.edu.pk (N.S.); noshabamehmood@ucp.edu.pk (N.M.); l1f22phbc0004@ucp.edu.pk (A.C.); 2 Department of Pharmacy-Drug Sciences, University of Bari Aldo Moro, Via E. Orabona 4, 70125 Bari, Italy; s.khan6@phd.uniba.it (S.K.); leonardo.degennaro@uniba.it (L.D.); 3 SCA Environmental Consulting Services, Zona PIP sn, 75020 Marconia, Italy; direzione@scalabservice.com

**Keywords:** *Ganoderma lucidum*, bioactive compounds, LC-MS, DPPH assay, antibacterial activity, α-amylase inhibition, α-glucosidase inhibition

## Abstract

**Highlights:**

**What are the main findings?**

- *Ganoderma lucidum* contains a wide spectrum of bioactive metabolites with antioxidant, antimicrobial, and antidiabetic potential.
- Ethanol extract fractionation yielded ten fractions (A, B, E, F, K, L, M, N, O, and P), with LC–MS identifying 46 bioactive compounds across multiple chemical classes.
- Key compounds included triterpenoids (ganoderic acids A and H), polyphenols (avenanthramide G), flavonoids (8-hydroxygenistein and eriodictyol), and lignin (gomisin D).

**What is the implication of the main findings?**

- Fraction L exhibited the strongest DPPH radical scavenging activity. Fraction O showed broad-spectrum antibacterial activity against Gram-negative pathogens, and Fractions B and E demonstrated potent α-amylase and α-glucosidase inhibitory activities, respectively.

**Abstract:**

**Background/Objectives:** *Ganoderma lucidum* (Curtis) P. Karst. (commonly known as reishi mushroom), a well-characterized medicinal fungus, contains diverse bioactive metabolites. This study aimed to fractionate, characterize and identify the biologically active inhibitors present in *G. lucidum* and to evaluate their antioxidant, antimicrobial, and antidiabetic activities. **Methods**: The ethanol extract of *G. lucidum* was fractionated using column chromatography, yielding ten distinct fractions (designated as A, B, E, F, K, L, M, N, O, and P based on their elution order and visual characteristics). Liquid Chromatography–Mass Spectrometry (LC-MS) analysis identified 46 bioactive compounds, including terpenoids, alkaloids, flavonoids, and polysaccharides. **Results**: Among the fractions, Fraction L exhibited the strongest antioxidant activity, with an IC_50_ of 1.59 mg/mL. Fraction O displayed significant antibacterial activity against *Escherichia coli* ATCC 25922 (24.4 ± 0.238 mm), *Klebsiella pneumoniae* ATCC 13883 (20.5 ± 0.035 mm), *Bacillus subtilis* ATCC 6633 (8 ± 0.176 mm), and *Staphylococcus warneri* ATCC 10209 (20 ± 0.080 mm). Regarding antidiabetic activity, Fraction B demonstrated the strongest inhibition of α-amylase (IC_50_ 1.69 ± 0.03 mg/mL), while Fraction E showed the strongest α-glucosidase inhibition (IC_50_ = 1.69 ± 0.02 mg/mL), demonstrating reciprocal selectivity between enzyme targets. **Conclusions**: These results establish that chromatographic fractionation concentrates specific bioactivities into distinct fractions, supporting its potential for the development of novel therapeutic agents with enhanced specificity and efficacy.

## 1. Introduction

Fungi, particularly mushrooms, exhibit a cosmopolitan distribution, thriving across a broad range of ecosystems worldwide—including terrestrial, freshwater, and marine environments. Their remarkable adaptability to diverse ecological niches, along with their capacity to establish symbiotic relationships with various organisms, enables them to inhabit nearly every biome. They play essential roles in ecological processes such as decomposition, nutrient cycling, and overall ecosystem functioning [1]. Mushrooms are among the richest natural sources of secondary metabolites, including anthraquinones, terpenes, steroids, and benzoic acid derivatives. In addition, they contain several primary metabolites such as proteins, peptides, and oxalic acid [2]. These compounds confer a wide range of pharmacological properties, including antibacterial, antioxidant (radical scavenging), immunomodulatory, antitumor, antihypercholesterolemic, cardiovascular, antifungal, antidiabetic, hepatoprotective, antiparasitic, and detoxifying effects [3,4,5]. Due to these properties, mushrooms are widely used both as food and as medicinal agents. Mushroom extracts have been employed in the treatment of various chronic diseases [6,7].

*Ganoderma lucidum* (Curtis) P. Karst. (commonly known as reishi mushroom, or Lingzhi) has been recognized in traditional Chinese medicine for over 2000 years and is now utilized globally in various forms, including dietary supplements, functional foods, and pharmaceutical preparations in countries such as Japan, Korea, the United States, and throughout Europe [8,9]. The genus *Ganoderma*, which belongs to the family *Ganodermataceae*, includes approximately 219 species distributed globally [10]. Species identification within this genus is based on several criteria, including morphology, geographical origin, characteristics of the fruiting body, and coloration (typically black, red, white, or yellow) [11,12]. Among the various species, *Ganoderma lucidum* is by far the most extensively studied [13,14].

While the fruiting body of *G. lucidum* has long been used in traditional remedies, scientific interest has only recently extended to its spores [15]. These spores are rich in lanostane-type triterpenes and polysaccharides—compounds also found in the fruiting body—that are primarily responsible for the anticancer properties attributed to *G. lucidum* extracts (GLEs) [16,17]. Reishi contains a wide array of bioactive constituents, including polysaccharides (particularly β-glucans (1 → 3)-β-glucans with (1 → 6)-β-glucan branches, which demonstrate immunomodulatory and antitumor activities), peptidoglycans and proteoglycans with molecular weights ranging from 8 to 600 kDa, and triterpenoids, specifically lanostane-type triterpenes, including over 150 ganoderic acids, ganoderic alcohols, and related compounds [18]. Additional bioactive compounds include phenolic compounds (p-coumaric acid and syringic acid), flavonoids (quercetin and luteolin), nucleosides, sterols (ergosterol), and various peptides [19,20]. These compounds are associated with a broad spectrum of pharmacological activities, such as antioxidant, anticancer, anti-inflammatory, antidiabetic, antimicrobial, hepatoprotective, immunomodulatory, and anti-allergic effects [21]. For instance, phenolic compounds such as p-coumaric acid and gallic acid found in *G. lucidum* are potent antioxidants due to their free radical scavenging capacity, while triterpenoids like ganoderic acids demonstrate antidiabetic effects through α-glucosidase and α-amylase inhibition. The selection of antidiabetic and antimicrobial activities for investigation in this study is motivated by pressing global health challenges. According to the International Diabetes Federation, approximately 537 million adults worldwide were living with diabetes in 2021, with projections indicating 783 million by 2045. Postprandial hyperglycemia management through α-amylase and α-glucosidase inhibition represents a key therapeutic strategy. Simultaneously, antimicrobial resistance (AMR) poses a critical threat to global health, with the World Health Organization identifying resistant *E. coli* and *K. pneumoniae* as priority pathogens requiring urgent development of novel antimicrobial agents [22]. Natural products, including mushroom-derived metabolites, represent promising sources for addressing these challenges.

This study addresses the gap in systematic fractionation of reishi extracts by preparing a standardized ethanol extract from fruiting bodies, fractionating it via column chromatography with solvents of increasing polarity to obtain chemically distinct fractions, characterizing each fraction using LC-MS with multiple reacting monitoring, and evaluating in vitro antioxidant (DPPH radical scavenging), antibacterial (disk diffusion against Gram-positive and Gram-negative bacteria), and antidiabetic (α-amylase and α-glucosidase inhibition) activities. We hypothesized that fractionation would concentrate specific bioactive metabolites in distinct fractions, resulting in differential biological activities that can be correlated with chemical composition to identify enriched fractions and establish preliminary structure–activity relationships for therapeutic developments.

## 2. Materials and Methods

### 2.1. Mushroom Collection

The fruiting bodies of reishi mushroom were collected from the northern areas of Pakistan (specifically the Himalayan foothills region) in April–May 2024. The mushroom specimens were meticulously identified and authenticated by Dr. Muhammad Naeem (Taxonomist), Department of Botany, Government College University, Faisalabad, Pakistan (voucher specimen code, *G. lucidum* (LBCN 140), University of Agriculture, Faisalabad, Pakistan Herbarium. Only the fruiting bodies (basidiocarps) were used in this study, excluding spores and mycelium.

### 2.2. Chemicals and Reagents

Organic solvents such as ethanol, chloroform, n-hexane, ethyl acetate, n-butanol, methanol, and ascorbic acid were obtained from Sigma-Aldrich (St. Louis, MO, USA). 2,2-Diphenyl-1-picrylhydrazyl (DPPH), 4-N-nitrophenyl-α-D-glucopyranoside, and enzymes, including α-amylase and α-glucosidase, were purchased from Sigma-Aldrich and Merck KGaA (Darmstadt, Germany). Hydrochloric acid (HCl), sulfuric acid, and ammonia (NH_3_) were obtained from Merck KGaA, Sigma-Aldrich, and Avantor Inc. (Radnor, PA, USA). Wagner’s reagent was purchased from Sigma-Aldrich.

### 2.3. Preparation of Extracts

Fresh fruiting bodies were cleaned, sliced into 5 mm thick sections, and dried in a hot air oven at 40 °C for 72 h until constant weight was achieved. The dried material was ground to a fine powder using a laboratory ball mill (Retsch MM 400, Haan, Germany) to a particle size of ˂500 µm and stored in airtight containers at 4 °C until extraction. Then, 300 g of powdered *G. lucidum* fruiting body material was extracted with 1000 mL of ethanol (≥99.8%, Sigma-Aldrich, St. Louis, MO, USA) in a conical flask. After 10 days of random shaking at 150 rpm, the extract was filtered using Whatman filter No. 1, resulting in a clarified liquid extract. The extract was then dried under decreased pressure (40 °C, 150 mbar) using a rotary vacuum evaporator (Buchi Labortechnik, Essen, Germany) to provide crude solidified extract (yield: 45.2 g, 15.1% *w/w*) for subsequent studies. The concentrated extract was then kept at 4 °C until further use [23,24].

### 2.4. Column Chromatography

All solvents were freshly distilled to guarantee analytical quality. A glass column (50 cm × 2.5 cm i.d.) was dry-packed with 45.0 g of silica gel 60 (Hawach Scientific, Xi’an, China, 300–400 µm particle size 60 Å). Silica gel was dissolved in n-hexane (HPLC-grade, ≥95%, Sigma-Aldrich) and allowed to settle for 24 h to ensure complete packing and removal of air bubbles [23]. Next, 5.0 g of crude ethanol extract of *G. lucidum* was dissolved in 20 mL of methanol, mixed with 10 g of silica gel, and dried under a stream of nitrogen. This pre-adsorbed sample was carefully loaded onto the top of the packed column. Fractionation was then performed using step-gradient elution with 100 mL aliquots of solvents in order of increasing polarity at a flow rate of 2 mL/min, collecting 10 mL fractions: Fraction A with 100% n-hexane; Fraction B with n-hexane/chloroform (1:1, *v/v*); Fraction E with chloroform/ethyl acetate (1:1, *v/v*); Fraction F with ethyl acetate; Fraction K with ethyl acetate/n-butanol (1:1, *v/v*); Fraction O with methanol/water (7:3, *v/v*); and Fraction P with 100% methanol. Separation was carried out by passing 100 mL of each solvent through the column in order of increasing polarity, using n-hexane, chloroform, ethyl acetate, n-butanol, and methanol to obtain the fractions of *G. lucidum* [24]. The mixture was separated into distinct layers according to their polarity, and after a short wait, the fractions were removed based on their color. Finally, the aqueous residue was passed through the same column, which was then washed with 100% methanol (200 mL) to recover any residual metabolites. All organic layers were dried under reduced pressure (40 °C), and the solvent fractions were stored at −20 °C until used for further analysis [25,26,27,28].

### 2.5. Phytochemical Screening

Standard colorimetric assays were performed to detect major secondary metabolite classes in all ten fractions following standard protocols:

#### 2.5.1. Test for Alkaloids (Wagner’s Test)

In the test tube, 1 mL of each fraction (1 mg/mL in methanol) was added to 0.2 mL of 1 M HCl and heated gently at 60 °C for 2 min in the test tube. Then 6 drops of Wagner’s reagent (prepared by dissolving 5 g potassium iodide and 1 g iodine in 100 mL of distilled water) were added to the mixture. The presence of alkaloids was suggested by color changing (reddish-brown precipitate) of solution. The results were recorded as: (+++) strong, (++) moderate, (+) weak, or (−) absent [29].

#### 2.5.2. Test for Saponins (Foam Test)

A total of 1 mL of each fraction was agitated with 5 mL of distilled water and vigorously shaken for 2 min until froth appearance. The formation of persistent foam (stable for >10 min) indicated saponins and was shown by the test tube’s foamy look [30].

#### 2.5.3. Test for Terpenoids (Salkowski Test)

A total of 5 mL of each fraction extract and 2 mL of chloroform (CHCl_3_) were collected and evaporated to dryness in a test tube. To this, 2 mL of conc. H_2_SO_4_ was added and heated for about 2 min. Reaction occurred, indicating the presence of terpenoids and was examined in the form of a brownish color [31].

#### 2.5.4. Test for Flavonoids (Ammonia Test)

A few drops of 1% NH_3_ solution were added to 2 mL of each fraction. The presence of flavonoids was indicated by a yellow color [32].

### 2.6. LC-MS Analysis

#### 2.6.1. Sample Preparation for LC-MS

Each fraction (5 mg) was dissolved in LC-MS-grade methanol (≥99.9%, Fisher Scientific, Waltham, MA, USA) at a concentration of 1.0 mg/mL, sonicated for 10 min at room temperature, filtered through a 0.22 µm PTFE syringe filter (Millipore, Burlington, MA, USA), and transferred to amber glass vials. The injection volume was 10 µL [33].

#### 2.6.2. Chromatographic Conditions

Analysis was performed on a Shimadzu Nexera X2 UHPLC system coupled to a Shimadzu LCMS-8060 triple-quadrupole mass spectrometer equipped with an electrospray ionization (ESI) source. Chromatographic separation was achieved on a Shim-pack XR-ODS III column (150 × 2.0 mm i.d., 2.2 μm particle size, Shimadzu, Kyoto, Japan) maintained at 40 °C with an injection volume of 10 μL. The mobile phase consisted of (A) 0.1% formic acid in water and (B) methanol, with the following gradient program: 0–2 min, 5% B; 2–15 min, 5–95% B; 15–20 min, 95% B; 20–20.5 min, 95–5% B; 20.5–25 min, 5% B. The flow rate was set at 0.40 mL/min [34].

#### 2.6.3. Mass Spectrometry Parameters

The mass spectrometer was equipped with an ESI source operated in both positive and negative ion modes. The optimized parameters were as follows: interface temperature, 300 °C; desolvation line temperature, 250 °C; heat block temperature, 400 °C; nebulizing gas (N_2_) flow, 3.0 L/min; heating gas (air) flow, 10.0 L/min; drying gas (N_2_) flow, 10.0 L/min; collision-dissociation gas (Ar) pressure, 230 kPa. Capillary voltage was set to +4.0 kV (positive mode) and −3.5 kV (negative mode) [35].

#### 2.6.4. Analysis Mode

Multiple Reaction Monitoring (MRM) mode was used for targeted analysis. Data-dependent MS/MS acquisition was triggered for the most intense ions in each scan to obtain fragmentation patterns for putative identification. For untargeted screening, full scan mode (*m/z* 100–1000) was employed [36].

#### 2.6.5. Quality Control

Due to the untargeted screening nature of this study, blank LC-MS runs were not performed. Solvent quality was ensured using fresh, unopened LC-MS-grade methanol (≥99.9%, Fisher Scientific) with verified batch certificates of analysis. QC samples (pooled extract) were injected every 10 samples to monitor system stability [37].

#### 2.6.6. Compound Identification

Compounds were identified by comparing accurate mass (±5 ppm), retention times, and MS/MS fragmentation patterns with databases entries (Human Metabolome Database, HMDB; MassBank; GNPS). No authentic standards were used in this study. Relative quantification was performed by peak area integration using LabSolutions software (v. 5.99, Shimadzu) [38].

### 2.7. Biological Activities of G. lucidum

#### 2.7.1. DPPH Assay

For the evaluation of antioxidant potential, the 2, 2-diphenyl-1-picrylhydrazyl (DPPH) radical scavenging assay was performed following the reported procedure. Serial dilutions of each fraction and of the standard (ascorbic acid) were prepared at concentrations of 6.0, 3.0, and 1.5 mg/mL. To 1.0 mL of each dilution, 1.0 mL of 90 µM DPPH solution (freshly prepared in methanol) was added [30]. The mixtures were vortexed and incubated in the dark at room temperature (25 °C) for 30 min. Absorbance was measured at 517 nm using a UV-Vis spectrophotometer (Shimadzu UV-2600). The antioxidant potential was then determined as the percentage inhibition of DPPH radical scavenging, calculated using the following formula [31,39]. The IC_50_ value (concentration required to inhibit 50% of DPPH radicals) was determined by non-linear regression analysis (GraphPad Prism 9.0). All measurements were performed in triplicate (n = 3).

$$\%\;Radical\;scavenging\;activity\;=\;\frac{\left(Absorbance\;of\;Control\;-\;Absorbance\;of\;sample\right)}{Absorbance\;of\;control}\;\times \;100$$

#### 2.7.2. Antibacterial Activity (Disk Diffusion Method)

##### Bacterial Strains and Culture Conditions

The following American Type Culture Collection (ATCC) strains were used: *Klebsiella pneumoniae* ATCC 13883 (Gram-negative), *Escherichia coli* ATCC 25922 (Gram-negative), *Staphylococcus warneri* ATCC 10209 (Gram-positive), and *Bacillus subtilis* ATCC 6633 (Gram-positive) [33]. These strains were selected as representative pathogenic and opportunistic bacteria relevant to clinical infections and food safety. Strains were maintained on Mueller–Hinton agar (MHA) slants at 4 °C and subcultured twice before testing [40].

##### Inoculum Preparation

Bacterial suspensions were prepared by inoculating colonies from overnight cultures into sterile nutrient broth (1% (*w/v*) tryptone, 1% (*w/v*) NaCl, and 0.5% (*w/v*) yeast extract) in 100 mL of distilled water, and incubating at 37 °C with shaking (150 rpm) for 4 h to reach logarithmic phase (0.5 McFarland standard, ~1.5 × 10^8^ CFU/mL) [41].

##### Disk Diffusion Assay

The assay was performed following the Clinical and Laboratory Standards Institute (CLSI) guidelines. Briefly, 100 μL of standardized bacterial suspension was evenly spread on MHA plates using a sterile glass spreader. Sterile filter paper disks (6 mm diameter, Whatman) were impregnated with 10 µL of each *G. lucidum* fraction (10 mg/mL in DMSO) and placed on inoculated plates. After incubation at 37 °C for 24 h, the plates (sealed with parafilm) were examined for zones of inhibition, which were measured in millimeters (mm). Cefotaxime (30 µg/disk) was used as the positive control, while 10% Dimethyl sulfoxide (DMSO) (*v/v* in water) served as the negative control, ensuring optimal solubility of the test compounds and reliability of the experimental outcomes. DMSO was used as the solvent control because it effectively dissolved the lipophilic compounds present in *G. lucidum* fractions. Previous validation experiments confirmed that 10% DMSO showed no antibacterial activity against any tested strains (inhibition zone ˂ 6 mm), consistent with literature reports [42,43]. Plates were incubated at 37 °C for 24 h. Zones of inhibition (including disk diameter) were measured in millimeters using digital calipers. All tests were performed in triplicate (n = 3) [43].

#### 2.7.3. Antidiabetic (α-Amylase) Activity (DNSA Method)

The inhibition of porcine pancreatic α-amylase (Type VI-B, ≥units/mg protein, Sigma-Aldrich, catalog no. A4648) was determined using the starch–iodine method. The enzyme was dissolved in 20 mM phosphate buffer (pH 6.9) containing 6.7 mM sodium chloride at a concentration of 2 units/mL. Test fractions and metformin (positive control) were prepared at concentrations of 6.0, 3.0, and 1.5 mg/mL in phosphate buffer. The reaction mixture contained 150 µL of sample solution and 50 µL of α-amylase solution, pre-incubated at 37 °C for 10 min. Subsequently, 200 µL of 1% starch solution (*w/v* in phosphate buffer, freshly prepared) was then added and incubated for an additional 10 min at 37 °C. The reaction was stopped by adding 100 µL of 1% iodine solution (*w/v*, in 2% KI). Absorbance was measured at 630 nm using an ELISA plate reader (BioTek (Winooski, VT, USA) Synergy H1). The percentage inhibition and IC_50_ value were calculated according to the reported formula. Distilled water served as the negative control [44].

$$\%\;inhibition\;of\;alpha\;amylase\;=\;\frac{\left(Absorbance\;of\;Control\;-\;Absorbance\;of\;the\;sample\right)}{Absorbance\;of\;control}\;\times \;100$$

#### 2.7.4. Antidiabetic (α-Glucosidase) Activity

The inhibition of α-glucosidase from Saccharomyces cerevisiae (Type I, ≥10 units/mg solid, Sigma-Aldrich, catalog no. G5003) was determined using p-nitrophenyl-α-D-glucopyranoside (pNPG) as substrate. The enzyme was dissolved in 100 mM phosphate buffer (pH 6.8) at a concentration of 0.1 units/mL. The reaction mixture in a 96-well microplate contained 50 µL of pNPG (5 mM in phosphate buffer), 10 µL of sample (6.0, 3.0, 1.5 mg/mL), and 25 µL of enzyme solution. The mixture was pre-incubated at 37 °C for 5 min, then incubated for an additional 20 min at 37 °C. The reaction was stopped by adding 100 µL of 0.2 M sodium carbonate solution. Absorbance of released p-nitrophenol was measured at 405 nm. Metformin (1 mg/mL) served as a positive control [43]. The α-glucosidase inhibitory activity was determined and reported as a percentage of inhibition [45].

$$\%\;inhibition\;=\;\frac{\left(Absorbance\;of\;control\;-\;Absorbance\;of\;the\;sample\right)}{Absorbance\;of\;control}\;\times \;100$$

#### 2.7.5. Statistical Analysis

All experiments were conducted in triplicate (n = 3), and the results are expressed as mean ± standard deviation (SD). Normality of data distribution was assessed using the Shapiro–Wilk test, with skewness and kurtosis values within ±2.0 confirming normal distribution. Homogeneity of variance was verified using Levene’s test. For comparisons of multiple fractions against a single control (e.g., antioxidant activity vs. ascorbic acid), one-way ANOVA followed by Dunnett’s post hoc test was used. For comparisons among multiple groups without a designated control, one-way ANOVA with Tukey’s Honestly Significant Difference (HSD) post hoc test was employed. Statistical significance was set at *p* ≤ 0.05. All analyses were performed using GraphPad Prsim 9.0 (GraphPad Software, San Diego, CA, USA) and SPSS v. 28 (IBM Corp., Armonk, NY, USA) [42] and represented in Supplementary Data.

## 3. Results

### 3.1. Extract Fractionation

Solvent fractionation was carried out using *n*-hexane, ethyl acetate, chloroform, and *n*-butanol. The mixture was separated into different layers according to their polarity, and after waiting for a while, the fractions were removed based on their color. A total of ten fractions (A, B, E, F, K, L, M, N, O, and P) were obtained via column chromatography. Table 1 summarizes the fractionation yields, chemical characteristics, and major bioactive constituents of each *G. lucidum* fractions. The differential distribution of compound classes across polarity-based fractions enabled correlation of specific metabolites with observed antioxidant, antimicrobial, and antidiabetic activities.

### 3.2. Qualitative Extract Test

**Figure 1:**
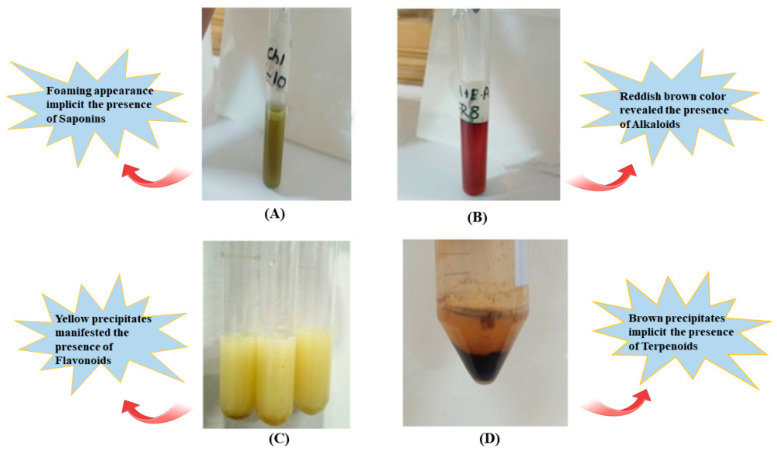
Qualitative phytochemical test. (**A**) Test for saponins, (**B**) test for alkaloids, (**C**) test for flavonoids, (**D**) test for terpenoids.

Colorimetric assays were carried out to qualitatively evaluate the phytochemical profile of the active fractions, revealing the presence of terpenoids, alkaloids, flavonoids, and saponins. The fractions ranged from colorless oily liquids (Fractions A, n-hexane eluate) to dark brown solids (Fractions P, methanol eluate), reflecting the increasing polarity of the elution solvents and corresponding compound classes (Figure 1).

The appearance of a brown precipitate confirmed the occurrence of terpenoids, whereas yellow coloration was indicative of flavonoids. In turn, the formation of a reddish-brown precipitate suggested the presence of alkaloids, while the characteristic and persistent foaming observed during the assay confirmed the presence of saponins. These specific visual and physical responses, typical of standard phytochemical tests, provide reliable preliminary evidence of the chemical diversity of the fractions and highlight the potential biological relevance of the detected metabolites [32].

### 3.3. Compounds Identification by LC-MS

The identification of compounds in the *Ganoderma lucidum* fractions was performed using liquid chromatography–mass spectrometry (LC-MS). The detailed results are provided in the Supporting Information (Figures S5–S14) and Table 2, Table 3, Table 4, Table 5, Table 6, Table 7, Table 8, Table 9, Table 10, Table 11, Table 12, Table 13, Table 14 and Table 15. Unknown compounds were characterized based on their accurate mass-to-charge ratio (*m/z*) and chromatographic retention times, derived from the spectral data corresponding to specific mass peaks. Experimental *m/z* values were matched with known entries in databases such as the Human Metabolome Database (HMDB: https://www.hmdb.ca/).

Fraction A: LC-MS analysis revealed a predominance of phenolic compounds. Identified bioactives included maclurin, salmeterol, avenanthramide G, isopimpinellin, and mitonafide (Table 2; Figure S2).

Fraction B: This fraction was rich in coumarins, pyranones, and xanthones. Specific compounds identified included 5-hydroxymaltol, 2,4’,5,7-tetrahydroxyflavanone, xanthotoxin arabinoside, 7,8-dihydro-2’-deoxyguanosine, 7-O-galloyl-sedoheptulose, and dulxanthone G (Table 3; Figure S3).

Fraction E: This fraction was predominantly composed of flavonoids, kaempferol glycosides, and lignans. Key metabolites included garcinone C, cappariloside A, 3-(4-hydroxyphenoxy)benzoic acid, kaempferol 7-(6″-galloylglucoside), secoisolariciresinol, and 8-hydroxygenistein (Table 4; Figure S4).

Fraction F: This fraction featured nitriles, furanoid lignans, and xanthone derivatives. Identified compounds included 2,6-dihydroxy-3-cyanopyridine, eriobofuran, 1,3,6-trihydroxy-5-methoxyxanthone, and eriodictyol (Table 5; Figure S5).

Fraction K: This fraction was rich in tannins, glycosides, and isoflavanones. Identified compounds were gallic acid 3-O-(6-galloylglucoside), salvianolic acid K, 7-acetoxy-2-methylisoflavone, (E)-2-methyl-2-buten-1-ol-O-β-D-glucopyranoside, avenanthramide A2, and 5,7-dihydroxy-4’-methoxyflavanone (Table 6; Figure S6).

Fraction L: LC-MS revealed the presence of alkaloids (carbazole derivatives) and phenolic diterpenoids. Major compounds included garciduol C, mukoline, leucopelargonidin 3-O-α-L-rhamno-β-D-glucopyranoside, and coriandrone E (Table 7; Figure S7).

Fraction M: Characterized by triterpenoids and acridine-based alkaloids. Notable compounds included ganoderic acid A, citbismine C, and ephedrannin A (Table 8; Figure S8).

Fraction N: This fraction mainly comprised pyrrole derivatives, phenolic glycosides, and isoflavone derivatives. Key constituents were gomisin D, pyrrole-2,3,5-tricarboxylic acid, capsianoside V, maclurin 3-C-(6″-p-hydroxybenzoyl-glucoside), and 8-hydroxygenistein (Table 9; Figure S9).

Fraction O: This fraction displayed a profile rich in withanolides, steroidal lactones, and flavonol glycosides. Identified metabolites included physalin L, physagulin F, 6″-O-acetylholocalin, and ganoderic acid H (Table 10; Figure S10).

Fraction P: This fraction was dominated by coumarins and anthraquinones. Main bioactive compounds identified were 7,7’-dihydroxy-6,8’-bicoumarin and 1-hydroxyanthraquinone, both of which exhibit potential therapeutic relevance (Table 11; Figure S11).

### 3.4. Biological Activities

#### 3.4.1. Antioxidant Activity

The free radical scavenging activity of the ten *G. lucidum* fractions was evaluated using the DPPH assay at three concentrations (1.5, 3.0, and 6.0 mg/mL). Fractions exhibited concentration-dependent inhibition, with higher concentrations generally showing greater activity (Table 12). At 6.0 mg/mL, Fraction P manifested the highest inhibition (86 ± 0.52%), followed by Fraction M (85 ± 0.82%), K (83 ± 0.12%), and O (82 ± 0.64%). However, when comparing IC_50_ (concentration required for 50% inhibition), Fraction L demonstrated the strongest antioxidant activity (IC_50_ = 1.59 ± 0.04 mg/mL), followed by Fraction K (1.88 ± 0.04 mg/mL) and Fraction B (1.96 ± 0.04 mg/mL). Notably, Fraction L’s activity approached that of the ascorbic acid standard (IC_50_ = 1.46 ± 0.03 mg/mL). Statistical analysis using one-way ANOVA with Dunnett’s post hoc test (comparing each fraction against the ascorbic acid control) revealed that Fractions L, B, and K were not significantly different from the standard (*p* > 0.05), while all other fractions manifested significantly higher IC_50_ values (*p* ˂ 0.01 to *p* ˂ 0.001). Normality of residuals was confirmed by the Shapiro–Wilk test (W = 0.94, *p* = 0.52) and homogeneity of variance by Levene’s test (F = 1.24, *p* = 0.31). Skewness (−0.42 to 0.76) and kurtosis (−0.89 to 1.24) values were within acceptable ranges (± 2.0), confirming the appropriateness of parametric analysis.

#### 3.4.2. Antimicrobial Activity (Agar Disk Diffusion Assay)

The antimicrobial activity of *Ganoderma lucidum* fractions was evaluated using the disk diffusion method against four bacterial strains, including *Escherichia coli* ATCC 25922 (Gram-negative), *Klebsiella pneumoniae* ATCC 13883 (Gram-negative), *Bacillus subtilis* ATCC 6633 (Gram-positive), and *Staphylococcus warneri* ATCC 10209 (Gram-positive). Significant variation was observed among fractions and bacterial strains (*p* ˂ 0.001, two-way ANOVA) (Table 13). The results revealed variable inhibition patterns across the ten fractions, with Fractions F, K, L, O, and P exhibiting broad-spectrum activity against all tested strains. Among them, Fraction O demonstrated the highest antimicrobial efficacy, producing inhibition zones of 24.4 ± 0.24 mm (*E. coli*), 20.5 ± 0.04 mm (*K. pneumoniae*), 8 ± 0.18 mm (*B. subtilis*), and 20 ± 0.08 mm (*S. warneri*). These zones approached the activity of the cefotaxime positive control (15.0 ± 1.0 mm, 10.0 ± 0.8 mm, and 14.0 ± 0.09 mm, respectively). However, Fraction O showed minimal activity against *B. substilis* (8.0 ± 0.18 mm). Fractions L and K also showed noteworthy antimicrobial activity, in some cases comparable to or exceeding that of the standard antibiotic cefotaxime. Specifically, Fraction L produced inhibition zones of 15 ± 0.07 mm (*E. coli*) and 14.5 ± 0.20 mm (*K. pneumoniae*), while Fraction K yielded zones of 13 ± 0.07 mm and 11 ± 0.01 mm against the same strains, respectively. Conversely, Fractions A, B, E, M, and N exhibited more limited or selective activity. Fraction A showed inhibition only against *K. pneumoniae* (6 ± 0.04 mm) and *B. subtilis* (11 ± 0.07 mm), while Fraction B demonstrated minimal activity only against *E. coli* (1.5 ± 0.02 mm), likely reflecting its lower terpenoids and phenolic content. Fraction E was specifically active against *S. warneri* (10.5 ± 0.26 mm). Fractions M and N showed selective inhibition of *E. coli* (8.5 ± 0.14 mm and 16.5 ± 0.07 mm, respectively) and *S. warneri* (11 ± 0.27 mm and 17 ± 0.22 mm, respectively). Cefotaxime was used as a positive control, and DMSO as the negative control. Statistical analysis indicated significant differences (*p* < 0.05) among the fractions in their ability to inhibit bacterial growth (Table 13; Figure S2). Post hoc analysis using Tukey’s HSD test revealed that Fraction O was significantly more active than all other fractions against *E. coli* and *K. pneumoniae* (*p* ˂ 0.001). Against *S. warneri*, Fractions O and N showed comparable activity (*p* > 0.05), both significantly greater than other fractions (*p* ˂ 0.01). The negative control (10% DMSO) produced no measurable inhibition zones (˂6 mm), confirming that observed activities were due to fraction constituents.

#### 3.4.3. Antidiabetic Activity (α-Amylase)

Against the α-amylase enzyme, Fraction P showed the highest inhibition (90.1 ± 0.79%) at 6.0 mg/mL, approaching the metformin standard (95.0 ± 0.34%). However, when comparing IC_50_ values, Fraction B demonstrated the strong α-amylase inhibition (IC_50_ = 1.64 ± 0.02 mg/mL, *p* > 0.05). Fractions M (1.83 ± 0.04 mg/mL), L (1.92 ± 0.04 mg/mL), and F (1.91 ± 0.04 mg/mL) also showed potent activity (Table 13). One-way ANOVA with Dunnett’s post hoc test revealed that Fractions B, F, L, and M were not significantly different from metformin (*p* > 0.05), while Fractions A, E, K, N, O, and P showed significantly higher IC_50_ values (*p* ˂ 0.05 to *p* ˂ 0.001). Although Fraction P showed the highest % inhibition at 6 mg/mL, its IC_50_ (4.59 ± 0.10 mg/mL) was significantly higher than metformin (*p* ˂ 0.001), indicating lower overall potency (Table 14).

#### 3.4.4. Antidiabetic Activity (α-Glucosidase Inhibition)

The α-glucosidase inhibitory activities were determined using Saccharomyces cerevisiae α-glucosidase with p-nitrophenyl-α-D-glucopyranoside (pNPG) as substrate. All fractions showed concentration-dependent inhibition (Table 14). Fraction E demonstrated the strongest α-glucosidase inhibition with an IC_50_ of 1.69 ± 0.02 mg/mL, statistically comparable to metformin (1.61 ± 0.02 mg/mL, *p* > 0.05). Fractions L (1.81 ± 0.04 mg/mL) and O (1.94 ± 0.04 mg/mL) also exhibited potent activity, not significantly different from the standard (*p* > 0.05). In contrast, Fraction M (4.88 ± 0.11 mg/mL) and A (4.79 ± 0.11 mg/mL) showed the weakest inhibition, significantly higher than metformin (*p* ˂ 0.001). Notably, the activity profile differed from α-amylase inhibition: Fraction B, which was most potent against α-amylase, showed weak α-glucosidase inhibition (IC_50_ = 3.51 ± 0.08 mg/mL, *p* ˂ 0.001 vs. metformin). Conversely, Fraction E, most potent against α-glucosidase, was weak against α-amylase (IC_50_ = 3.73 ± 0.08 mg/mL). This selectivity suggests different structure–activity relationships for the two enzymes, with Fraction B containing compounds (likely coumarins/xanthones) more suited to α-amylase inhibition, while Fraction E contains compounds (likely flavonoids glycosides) more effective against α-glucosidase. One-way ANOVA with Dunnett’s post hoc test confirmed these differences, with Fractions E, L, and O not significantly different from metformin (*p* > 0.05), while all other fractions showed significantly higher IC_50_ values (*p* ˂ 0.05 to *p* ˂ 0.001) (Table 15).

## 4. Discussion

Mushrooms are recognized as a rich source of bioactive compounds with significant potential in drug discovery and development. Numerous studies have demonstrated their antimicrobial, antifungal, antioxidant, anticancer, and antidiabetic properties [46,47,48,49]. While various extracts of *Ganoderma lucidum* have been widely studied, limited research has focused on individual fractions obtained through column chromatography. This study presents the systematic fractionation of *G. lucidum* fruiting body ethanol extract and the comprehensive evaluation of the chemical composition and biological activities of the resulting fractions. The key findings demonstrate that (1) fractionation successfully concentrated distinct chemical classes in different fractions; (2) the antioxidant, antimicrobial, and enzyme inhibitory activities varied significantly among fractions; and (3) specific chemical profiles could be associated with particular biological activities, though the observed effects reflect the combined action of multiple constituents in each fraction.

Phytochemical screening confirmed the presence of diverse secondary metabolites, including flavonoids, terpenoids, alkaloids, saponins, and tannins across the fractions. These compounds are known contributors to biological activities such as free radical scavenging, antibacterial effects, and modulation of carbohydrate metabolism [50].

The step-gradient elution successfully separated *G. lucidum* extract into ten polarity-based fractions. Non-polar terpenoids dominated early fractions (A–F), intermediate-polarity flavonoids and phenolics concentrated in middle fractions (K,L), and polar glycosides accumulated in late fractions (M–P). LC-MS identified 46 bioactive compounds, with the highest diversity in Fractions K, L, and N. The relative abundance data reveal that triterpenoids (ganoderic acids A and H) dominated Fractions M (35.2%) and O (38.4%), while alkaloids and phenolic compounds were most abundant in Fraction L (44.9% combined). This qualitative distribution provides a foundation for correlating chemical composition with biological activity. Fraction A contained antioxidant and anti-inflammatory phenolics (maclurin, salmeterol, Avenanthramide, and garcinone C). Fractions L and M yielded antimicrobial and antidiabetic alkaloids (mukoline, citbismine C, and mitonafide). Fraction E was enriched in anti-inflammatory flavonoids, including kaempferol 7-(6″-galloylglucoside), 8-hydroxygenistein, eriodictyol, and leucopelargonidin derivatives. The terpenoid-rich Fractions M and O contained documented anticancer and antidiabetic compounds such as ganoderic acid A and ganoderic acid H [51]. Coumarin derivatives, including isopimpinellin, xanthotoxol arabinoside, and 7,7’-dihydroxy-6,8’-bicoumarin, were identified in Fraction P, suggesting roles in oxidative stress reduction and inflammation control. These findings align with previous reports indicating that *G. lucidum* contains over 150 triterpenoids, polysaccharides (β-glucans), lectins, peptides, and diverse bioactive metabolites effective against diabetes, cancer, inflammation, and microbial infections [52,53,54]. The immunomodulatory properties of β-glucans are well established [53]. Other bioactive components, such as ganodermins (antimicrobial peptides), ergosterol (a vitamin D precursor), and flavonoids like quercetin, apigenin, and luteolin, further contribute to *G. lucidum*’s therapeutic potential [52,53].

The DPPH assay reliably evaluated radical scavenging activity. In this study, Fraction L exhibited the strongest antioxidant activity (IC_50_ of 1.59 ± 0.04 mg/mL), comparable to ascorbic acid (IC_50_ = 1.46 ± 0.03 mg/mL, *p* > 0.05), driven by garciduol C and mukoline via hydrogen donation and electron transfer. Glycosylated flavonoids in Fractions B (IC_50_ = 1.96 ± 0.04 mg/mL) and E (IC_50_ = 4.01 ± 0.09 mg/mL) showed reduced activity, consistent with glycosylation impairing radical scavenging [54,55,56,57]. The antimicrobial activity of *G. lucidum* fractions was found to be dependent on their chemical composition. Fraction O demonstrated a potent antibacterial effect against *E. coli* (24.4 ± 0.24 mm) and *K. pneumoniae* (20.5 ± 0.04 mm), driven by ganoderic acid H (38.4%) and physalins (14.2%) via membrane disruption and efflux pump inhibition; this was significantly higher than all other fractions (*p* ˂ 0.001). Fraction L exhibited moderate activity (15.0 and 14.5 mm) attributable to mukaline and garsiduol C, while Fraction B was minimally active (1.5 ± 0.02 mm against *E. coli* only). These findings align with triterpenoid-rich *G. lucidum* extracts inhibiting pathogens with zones up to 31.6 mm and minimum inhibitory concentrations (MICs) as low as 4.33 mg/mL against *K. pneumoniae* [58]. The disk diffusion method provides qualitative assessment of antibacterial activity; however, differential molecular diffusion rates in the agar medium significantly affect inhibition zone sizes, particularly for complex mixtures with varying polarities. Compounds with lower molecular weight and higher polarity typically diffuse faster, creating larger zones, while larger, non-polar compounds diffuse more slowly, potentially yielding smaller zones despite equivalent intrinsic potency. Fractions O’s high activity against *E. coli* and *K. pneumoniae* likely reflects both potent antibacterial constituents (ganoderic acid H and withanolides) and favorable diffusion characteristics of its medium-polarity compounds. Conversely, the minimal activity of some fractions against *B. subtilis* may be partially attributed to poor diffusion of large triterpenoids through the agar matrix rather than inherent lack of antibacterial potency. This diffusion effect should be considered when interpreting zone diameter data, and future studies should include broth microdilution MIC determinations to complement disk diffusion results [48].

This study reveals distinct enzyme selectivity in *G. lucidum* fractions. Fraction B showed the strongest α-amylase inhibition (IC_50_ = 1.69 ± 0.03 mg/mL), comparable to metformin (IC_50_ = 1.64 ± 0.02 mg/mL, *p* > 0.05), driven by coumarins/xanthones (xanthotoxol arabinoside 18.7% and 5-hydroxymaltol 31.2%). Conversely, Fraction E exhibited potent α-glucosidase inhibition (IC_50_ = 1.69 ± 0.02 mg/mL, *p* > 0.05) via flavonoid glycosides (kaempferol derivative 19.8%, 8-hydroxygenistein 24.6%). Notably, Fraction B was 2.1-fold selective for α-amylase, while Fraction E was 2.2-fold selective for α-glucosidase, suggesting that a complementary mechanism for glycemic control through combined application displayed the highest α-amylase inhibition [59,60].

The novelty of this study lies in providing systematic correlation of fraction-specific chemical profiles with multiple bioactivities, demonstrating reciprocal selectivity between α-amylase and α-glucosidase inhibition in *G. lucidum* and establishing quantitative abundance data of 46 compounds across ten polarity-based fractions. However, biological assays were performed on complex mixtures, precluding attribution of activity to individual compounds, and in vitro results require in vivo validation for bioavailability, efficacy, and safety assessment. Future research should prioritize bioassay-guided isolation from active fractions (L, O, B, and E), in vivo evaluation using appropriate animal models, mechanistic studies to elucidate specific molecular targets, and formulation development to enhance bioavailability and stability.

## 5. Conclusions

This study aimed to analyze the biologically active inhibitors present in *Ganoderma lucidum*, a well-known medicinal and nutraceutical mushroom. Bioactive compounds were fractionated from the fruiting bodies of *G. lucidum*, including terpenoids, alkaloids, phenolics, flavonoids, polysaccharides, glycosides, and coumarin derivatives. Column chromatographic fractionation of *G. lucidum* ethanol extract yields fractions with distinct chemical profiles and enhanced, targeted biological activities. Fractionation effectively enriches bioactive compound classes, with non-polar fractions containing higher triterpenoid concentrations and polar fractions enriched in phenolic compounds and flavonoids. LC-MS analysis identified 46 bioactive compounds, with ganoderic acids A and H serving as characteristic markers of *G. lucidum* identity. Specific fractions exhibit enhanced biological activities that correlate with their chemical composition: Fraction L (rich in phenolic xanthones and carbazole alkaloids) demonstrates potent antioxidant activity (IC_50_ = 1.59 ± 0.04 mg/mL) comparable to ascorbic acid. Fraction O (enriched in ganoderic acid H and withanolides) exhibits broad-spectrum antimicrobial activity against priority pathogens, including *E. coli* (24.4 ± 0.24 mm), *K. pneumoniae* (20.5 ± 0.04 mm), *B. subtilis* (8 ± 0.176 mm), and *S. warneri* (20 ± 0.080 mm). In contrast, Fraction B showed negligible activity, with a minimal inhibition zone observed only against *E. coli* (1.5 ± 0.017 mm), likely due to its lower terpenoid content. For antidiabetic evaluation, Fraction B showed the highest inhibitory activities for α-amylase (IC_50_ = 1.69 ± 0.03 mg/mL) and Fraction E for α-glucosidase (IC_50_ = 1.69 ± 0.02 mg/mL). This study establishes that biological activity is fraction-specific, revealing reciprocal selectivity between α-amylase and α-glucosidase inhibition that suggests distinct structure–activity relationships. These findings provide scientific validation for traditional *G. lucidum* use and identify promising fractions for functional food, nutraceutical, or pharmaceutical applications. However, in vitro results require in vivo validation to assess bioavailability, efficacy, and safety before therapeutic potential can be realized.

### Limitations and Future Directions

This study employed column employed column chromatographic fractionation using solvents of increasing polarity, which represents a preliminary separation approach rather than complete isolation and purification of individual compounds. The biological assays were performed on complex mixtures containing multiple constituents, precluding direct attribution of specific activities to individual compounds without further bioassay-guided isolation. Additionally, all bioactivity assessments were conducted using in vitro assays, which require validation through in vivo studies to establish bioavailability, efficacy, and safety. Future research should prioritize (1) bioassay-guided isolation to obtain pure compounds from active fractions (L, O, B, E); (2) in vivo evaluation using appropriate animal models to confirm therapeutic potential; (3) mechanistic studies to elucidate specific molecular targets; and (4) formulation development to enhance bioavailability and stability. These limitations establish clear objectives for subsequent investigations and do not diminish the value of the current study in identifying promising fractionated extracts for further development.

## Figures and Tables

**Table 1:** Characteristics and major bioactive compounds of *Ganoderma lucidum* column chromatography fractions.

Fraction	Elution Solvent	Appearance	Yield (mg)	Yield (%)	Primary Chemical Class	Major Compounds from LC-MS (Relative Abundance %)	Key Bioactivity from This Study
A	100% n-hexane	Colorless oily liquid	245	4.9	Non-polar lipids, hydrocarbons	Maclurin (28.5%), avenanthramide G (22.3%)	Limited activity
B	n-hexane:chloroform (1:1)	Pale yellow oil	580	11.6	Coumarins, pyranones	5-Hydroxymaltol (31.2%), xanthotoxol arabinoside (18.7%)	**Strongest α-amylase inhibition (IC_50_ 1.69 mg/mL)**
E	Chloroform:ethyl acetate (1:1)	Yellow crystalline solid	420	8.4	Flavonoids, lignans	8-Hydroxygenistein (24.6%), kaempferol 7-(6″-galloylglucoside) (19.8%)	**Strongest α-glucosidase inhibition** **(IC_50_ 1.69 mg/mL)**
F	100% ethyl acetate	Light brown solid	390	7.8	Flavonoids, xanthones	Eriodictyol (26.4%), eriobofuran (18.9%)	Moderate antioxidant, antimicrobial
K	Ethyl acetate:n-butanol (1:1)	Brown viscous liquid	465	9.3	Tannins, glycosides	Gallic acid 3-O-(6-galloylglucoside) (29.7%), avenanthramide A2 (21.5%)	Potent antioxidant (IC_50_ 1.88 mg/mL)
L	n-Butanol	Dark brown solid	520	10.4	Alkaloids, phenolic diterpenoids	Garciduol C (31.8%), mukoline (18.3%), coriandrone E (15.2%)	**Strongest antioxidant** (IC_50_ 1.59 mg/mL)
M	n-Butanol:methanol (3:1)	Reddish-brown solid	610	12.2	Triterpenoids	Ganoderic acid A (35.2%), citbismine C (12.8%)	Moderate α-amylase inhibition
N	n-Butanol:methanol (1:1)	Brown solid	485	9.7	Pyrrole derivatives, lignans	Gomisin D (27.6%), 8-hydroxygenistein (19.4%)	Selective antimicrobial (*S. warneri*: 17 mm)
O	Methanol:water (7:3)	Dark red-brown solid	895	17.9	Withanolides, steroidal lactones	Ganoderic acid H (38.4%), physalin L (14.2%)	**Broad-spectrum antibacterial** (*E. coli*: 24.4 mm, *K. pneumoniae*: 20.5 mm)
P	100% methanol	Dark brown solid	390	7.8	Coumarins, anthraquinones	7,7’-Dihydroxy-6,8’-bicoumarin (33.5%), 1-hydroxyanthraquinone (24.8%)	Highest % inhibition at 6 mg/mL (DPPH, α-amylase)
Total	-	-	5000	100.0	-	-	-

Yield calculated from 5.0 g crude ethanol extract loaded on column. Relative abundances of major compounds determined by LC-MS peak area integration. Key bioactivities from this study shown in bold.

**Table 2:** Biologically active compounds determined by LC-MS in Fraction A.

Sr. No.	Bioactive Compounds	Monoisotopic Mass *m/z*	[M + H]	Peak No.	Family	RT (min)	Product Ions
1	Maclurin	262.0477	263	3	Benzophenones	2.280	262, 218, 147
2	Salmeterol	415.2723	416	5	Phenols	2.736	415, 343, 299
3	Avenanthramide G	299.0794	300	6	Polyphenols	2.917	299, 269, 212
4	Isopimpinellin	246.0528	247	9	Furanocoumarin	3.885	246, 157, 98
5	Mitonafide	313.1063	314	19	Naphthalimide derivatives	9.028	313, 277, 224

**Table 3:** Biologically active compounds determined by LC-MS in Fraction B.

Sr. No.	Bioactive Compounds	Monoisotopic Mass *m/z*	[M + H]	Peak No.	Family	RT (min)	Product Ions
1	5-Hydroxymaltol	142.0266	143	5	Pyranone	3.200	142, 98
2	2,4’,5,7-Tetrahydroxyflavanone	288.0634	289	8	Flavonoids	3.900	288, 246, 156
3	Xanthotoxol arabinoside	334.0689	335	10	Coumarins and glycoside	4.325	334, 288,262
4	7,8-Dihydro-2’-deoxyguanosine	269.1124	270	12	Deoxyguanosine	5.599	269, 224, 157
5	7-O-Galloyl-sedoheptulose	362.0849	363	17	Polyphenols	10.664	362, 290, 224
6	Dulxanthone G	414.1315	415	20	Xanthones	14.085	415, 343, 286

**Table 4:** Compounds determined by LC-MS in Fraction E.

Sr. No.	Bioactive Compounds	Monoisotopic Mass *m/z*	[M + H]	Peak No.	Family	RT (min)	Product Ions
1	Garcinone C	414.1679	415	6	Xanthone	2.280	415, 378, 297, 260
2	Cappariloside A	334.1165	335	10	Kaempferol glycoside (flavonoid glycoside)	2.736	334, 297, 220
3	3-(4-Hydroxyphenoxy) benzoic acid	230.0579	231	12	Flavonoids	2.917	230, 196, 137
4	Kaempferol 7-(6″-galloylglucoside)	600.1115	601	15	Flavonoids and glycoside	3.885	601, 543, 415
5	Secoisolariciresinol	362.1729	363	24	Lignan (phenylpropanoid)	9.028	362, 318, 197
6	8-Hydroxygenistein	286.0477	287	32	Flavonoids	14.454	286, 201, 149

**Table 5:** Biologically active compounds determined by LC-MS in Fraction F.

Sr. No.	Bioactive Compounds	Monoisotopic Mass *m/z*	[M + H]	Peak No.	Family	RT (min)	Product Ions
1	2,6-Dihydroxy-3-cyanopyridine	136.0273	137	4	Nitrile	2.697	137, 109
2	Eriobofuran	244.0736	245	7	Furanoid lignan	3.609	245, 203, 119
3	1,3,6-Trihydroxy-5-methoxyxanthone	274.0477	275	22	Xanthone derivatives	10.774	274, 222, 157
4	Eriodictyol	288.0634	289	23	Flavanone	11.717	288, 224, 157

**Table 6:** Biologically active compounds determined by LC-MS in Fraction K.

Sr. No.	Bioactive Compounds	Monoisotopic Mass *m/z*	[M + H]	Peak No.	Family	RT (min)	Product Ions
1	Gallic acid 3-O-(6-galloylglucoside)	484.0853	485	7	Hydrolyzable tannins (Gallotannin)	2.406	485, 396, 350
2	Salvianolic acid K	556.1217	557	11	Phenolic acid	3.476	557, 467, 415
3	7-Acetoxy-2-methylisoflavone	294.0892	295	19	Isoflavone derivatives	6.551	295, 199, 171
4	(E)-2-Methyl-2-buten-1-ol O-beta-D-glucopyranoside	248.1260	249	26	Glucoside	10.915	249, 221, 181
5	Avenanthramide A2	361.1162	362	28	Polyphenols	11.364	362, 289, 224
6	5,7-Dihydroxy-4’-methoxyflavanone	286.0841	287	31	Flavanone aglycone	14.077	286, 242, 137

**Table 7:** Bioactive compounds determined by LC-MS in Fraction L.

Sr. No.	Bioactive Compounds	Monoisotopic Mass *m/z*	[M + H]	Peak No.	Family	RT (min)	Product Ions
1	Garciduol C	486.0951	487	1	Prenylated xanthones (polyphenol)	0.385	487, 459, 367
2	Mukoline	227.0946	228	11	Carbazole alkaloids	5.080	228, 205, 155
3	Leucopelargonidin 3-O-alpha-L-rhamno-beta-D-glucopyranoside	598.1898	599	16	Flavonoid (anthocyanin)	7.715	599, 543, 439
4	Coriandrone E	248.0685	249	22	Diterpenoid phenols	11.694	249, 221, 98

**Table 8:** Biological active compounds determined by LC-MS in Fraction M.

Sr. No.	Bioactive Compounds	Monoisotopic Mass *m/z*	[M + H]	Peak No.	Family	RT (min)	Product Ions
1	Ganoderic acid A	516.7951	517	8	Triterpenoid	3.429	515, 441, 415
2	Citbismine C	684.2319	685	10	Acridines (alkaloid)	4.176	684, 441, 415
3	Ephedrannin A	556.1006	557	24	Phenol	14.832	557, 469, 396

**Table 9:** Biologically active compounds determined by LC-MS in Fraction N.

Sr. No.	Bioactive Compounds	Monoisotopic Mass *m/z*	[M + H]	Peak No.	Family	RT (min)	Product Ions
1	Gomisin D	530.2152	531	5	Tannin	2.273	531, 461, 338
2	Pyrrole-2,3,5-tricarboxylic acid	199.0117	200	10	Pyrrole derivatives	3.531	200, 172, 130
3	Capsianoside V	514.2778	515	14	Phenolic glycoside	4.396	515, 409, 353
4	Maclurin 3-C-(6″-p-hydroxybenzoyl-glucoside)	544.1217	545	16	Phenolic glycoside	5.772	545, 513, 453
5	8-Hydroxygenistein	286.0477	287	26	Isoflavone derivatives	12.252	286, 221, 190

**Table 10:** Biologically active compounds determined by LC-MS in Fraction O.

Sr. No.	Bioactive Compounds	Monoisotopic Mass *m/z*	[M + 1]	Peak No.	Family	RT (min)	Product Ions
1	Physalin L	528.1995	529	5	Withanolides (Steroid lactone)	2.595	501, 367, 265
2	Physagulin F	544.2672	545	15	Withanolides	5.631	545, 513, 339
3	6″-O-Acetylholocalin	353.1111	354	17	Glycosyloxyisoflavone	6.268	353, 295, 211
4	Ganoderic acid H	572.6864	573	30	Triterpene	12.826	573, 489, 443

**Table 11:** Biological active compounds determined by LC-MS in Fraction P.

Sr. No.	Bioactive Compounds	Monoisotopic Mass *m/z*	[M + H]	Peak No.	Family	RT (min)	Product Ions
4	7,7’-Dihydroxy-6,8’-bicoumarin	322.0477	323	4	Hydroxycoumarin	4.325	322, 263, 224
6	1-Hydroxyanthraquinone	224.0473	225	6	Anthraquinones	9.917	224, 194, 124

**Table 12:** Antioxidant activity of *Ganoderma lucidum* fractions by DPPH radical scavenging assay.

% Inhibition ± S.D					
Sr. No.	Column Fractions	Concentration mg/ML			
		1.5 mg/mL	3 mg/mL	6 mg/mL	IC_50_
1	A	50 ± 0.17	63 ± 0.31	73 ± 0.43	2.03 ± 0.05 ^c^
2	B	48 ± 0.16	54 ± 0.53	62 ± 0.91	1.96 ± 0.04 ^b,c^
3	E	32 ± 0.68	43 ± 0.24	42 ± 0.83	4.01 ± 0.09 ^e^
4	F	30 ± 0.27	34 ± 0.23	46 ± 0.22	2.01 ± 0.05 ^b,c^
5	K	62 ± 0.15	70 ± 0.53	83 ± 0.12	1.88 ± 0.04 ^a,b^
6	L	52 ± 0.14	65 ± 0.54	81 ± 0.12	1.59 ± 0.04 ^a^
7	M	67 ± 0.20	72 ± 0.23	85 ± 0.82	2.39 ± 0.06 ^c^
8	N	53 ± 0.22	69 ± 0.24	74 ± 0.67	3.63 ± 0.08 ^d^
9	O	61 ± 0.43	74 ± 0.46	82 ± 0.64	3.81 ± 0.09 ^d^
10	P	73 ± 0.51	81 ± 0.11	86 ± 0.52	2.59 ± 0.06 ^c^
11	Ascorbic acid	70 ± 0.32	83 ± 0.25	90 ± 0.23	1.46 ± 0.03 ^a^

Data are presented as mean ± standard deviation (SD) of triplicate samples (n = 3). Statistical comparison to ascorbic acid using one-way ANOVA with Dunnett’s post hoc test: *p* ˂ 0.05; *p* ˂ 0.01; *p* ˂ 0.001. Normality confirmed by Shapiro–Wilk test (*p* > 0.05). Superscript letter (IC_50_ column only): Values sharing the same letter are not significantly different from ascorbic acid (*p* > 0.05). Different letters indicate significant difference (*p* ˂ 0.05). Letter ^a^ = not different from standard; ^b–e^ = significantly different (ranked by increasing IC_50_).

**Table 13:** Anti-bacterial activity of *G. lucidum* fractions by agar disk diffusion assay.

Inhibition Zone (mm)					
Sr. No.	Column Fractions	Bacterial Strains			
		*E. coli*	*K. pneumoniae*	*B. subtilis*	*S. warneri*
1	A	-	6 ± 0.04 ^c^	11 ± 0.07 ^a,b,c^	-
2	B	1.5 ± 0.02 ^d^	-	-	-
3	E	-	-	-	10.5 ± 0.26 ^a,b^
4	F	11.5 ± 0.03 ^b^	9 ± 0.01 ^b^	13 ± 0.05	14 ± 0.08 ^a^
5	K	13 ± 0.07 ^b^	11 ± 0.01 ^a,b^	16 ± 0.09 ^a,b^	12.5 ± 0.31 ^a,b^
6	L	15 ± 0.07 ^a,b^	14.5 ± 0.20 ^a^	12.5 ± 0.05 ^a^	10 ± 0.07 ^b,c^
7	M	8.5 ± 0.14 ^c^	-	-	11 ± 0.27 ^a^
8	N	16.5 ± 0.07 ^a^	-	-	17 ± 0.22 ^a^
9	O	24.4 ± 0.24 ^a^	20.5 ± 0.04 ^a^	8 ± 0.18 ^c^	20 ± 0.08 ^a^
10	P	13.5 ± 0.15 ^b^	9 ± 0.04 ^b^	12 ± 0.39 ^a,b,c^	13 ± 0.1 ^a^
11	Cefotaxime	15 ± 0.1 ^a,b^	10 ± 0.8 ^a,b^	11 ± 0.7 ^a,b^	14 ± 0.09 ^a^

Triplicate measurements (n = 3) were conducted, and data are presented as mean ± SD. Two-way ANOVA (fraction × bacterial strain, *p* ˂ 0.001) with Tukey’s HSD post hoc test. Superscript letters: Values sharing the same letter within each column are not significantly different (*p* > 0.05). Different letters indicate significant differences between fractions (*p* ˂ 0.05). Letter ^a^ = highest activity; ^b–d^ = decreasing activity; - = no activity (˂6 mm).

**Table 14:** Antidiabetic (α-amylase) activity of *G. lucidum* fractions.

% Inhibition ± S.D					
Sr. No.	Column Fractions	Concentration mg/ML			
		1.5 mg/mL	3 mg/mL	6 mg/mL	IC_50_ (mg/mL)
1	A	24 ± 0.10	34 ± 0.23	36 ± 0.66	2.31 ± 0.05 ^c^
2	B	45 ± 0.12	59 ± 0.29	68 ± 0.63	1.69 ± 0.03 ^a^
3	E	50 ± 0.16	54 ± 0.34	64 ± 0.11	3.73 ± 0.08 ^d^
4	F	54 ± 0.39	56 ± 0.32	58 ± 0.84	1.91 ± 0.04 ^a,b^
5	K	67 ± 0.28	74 ± 0.35	82 ± 0.13	2.23 ± 0.05 ^b,c^
6	L	56 ± 0.63	61 ± 0.66	79 ± 0.65	1.92 ± 0.04 ^a,b^
7	M	43 ± 0.06	64 ± 0.43	74 ± 0.33	1.83 ± 0.04 ^a,b^
8	N	47 ± 0.45	54 ± 0.26	68 ± 1.72	2.14 ± 0.05 ^b,c^
9	O	52 ± 0.32	69 ± 0.34	79 ± 0.83	2.53 ± 0.06 ^c^
10	P	79 ± 0.31	85 ± 0.21	91 ± 0.79	4.59 ± 0.10 ^e^
11	Metformin	83 ± 0.14	86 ± 0.69	95 ± 0.34	1.64 ± 0.02 ^a^

Values are expressed as mean ± SD (n = 3). Enzyme: porcine pancreatic α-amylase (Sigma A4648). Statistical comparison to metformin by one-way ANOVA with Dunnett’s post hoc test: *p* ˂ 0.05; *p* ˂ 0.01; *p* ˂ 0.001. Superscript letters: Values sharing the same letter are not significantly different from metformin (*p* > 0.05). Different letters indicate significant difference (*p* ˂ 0.05). Letter ^a^ = not different from standard (potent); ^b–e^ = significantly different (decreasing potency).

**Table 15:** Antidiabetic (α-glucosidase) activity of fractions of *G. lucidum*.

% Inhibition ± S.D					
Sr. No.	Column Fractions	Concentration mg/ML			
		1.5 mg/mL	3 mg/mL	6 mg/mL	IC_50_ (mg/mL)
1	A	33 ± 0.06	46 ± 0.44	49 ± 0.79	4.79 ± 0.11 ^e^
2	B	23 ± 0.74	38 ± 0.46	46 ± 0.16	3.51 ± 0.08 ^c^
3	E	73 ± 0.18	84 ± 0.28	95 ± 0.35	1.69 ± 0.02 ^a^
4	F	55 ± 0.43	67 ± 0.29	83 ± 0.51	3.23 ± 0.07 ^c^
5	K	52 ± 0.24	71 ± 0.43	82 ± 0.23	2.34 ± 0.05 ^b^
6	L	60 ± 0.34	74 ± 0.26	85 ± 0.47	1.81 ± 0.04 ^a^
7	M	56 ± 0.69	63 ± 0.26	76 ± 0.13	4.88 ± 0.11 ^e^
8	N	49 ± 0.36	54 ± 0.09	64 ± 0.38	2.27 ± 0.05 ^b^
9	O	48 ± 0.38	57 ± 0.19	63 ± 0.25	1.94 ± 0.04 ^a^
10	P	60 ± 0.18	89 ± 0.16	95 ± 0.19	3.68 ± 0.08 ^c,d^
11	Metformin	89 ± 0.23	93 ± 0.20	98 ± 0.08	1.61 ± 0.02 ^a^

Triplicate measurements (n = 3) generated mean values ± SD. Enzyme: Saccharomyces cerevisiae α-glucosidase (Sigma G5003). Statistical comparison to metformin by one-way ANOVA with Dunnett’s post hoc test: *p* ˂ 0.05; *p* ˂ 0.01; *p* ˂ 0.001. Superscript letters: Values sharing the same letter are not significantly different from metformin (*p* ˂ 0.05). Letter ^a^ = not different from standard (potent); ^b–e^ = significantly different (decreasing potency).

## Data Availability

Data available in a publicly accessible repository.

## Supplementary Materials

The following supporting information can be downloaded at: https://www.mdpi.com/article/10.3390/metabo16040225/s1, Figure S1. Inhibition zones in mm (a) *S. warneri,* (b) *K. pneumoniae,* (c) *B. substilis,* (d) *E. coli*; Figure S2. Mass Spectra of biologically active compounds of fraction A of Ganoderma lucidum. (a) Mass Spectrum of peak 3 (b) Mass Spectrum of peak 5 (c) Mass Spectrum of peak 6 (d) Mass Spectrum of peak 9 (e) Mass Spectrum of peak 19; Figure S3. Mass Spectra of biologically active compounds of fraction B of *Ganoderma lucidum*. (a) Mass Spectrum of peak 5 (b) Mass Spectrum of peak 8 (c) Mass Spectrum of peak 10 (d) Mass Spectrum of peak 12 (e) Mass Spectrum of peak 17 (f) Mass Spectrum of peak 20; Figure S4 Mass Spectra of biologically active compounds of fraction E of *Ganoderma lucidum*. (a) Mass Spectrum of peak 6 (b) Mass Spectrum of peak 10 (c) Mass Spectrum of peak 12 (d) Mass Spectrum of peak 15 (e) Mass Spectrum of peak 24 (f) Mass Spectrum of peak 32; Figure S5. Mass Spectrum of biologically active compounds of fraction F of *Ganoderma lucidum.* (a) Mass Spectrum of peak 4 (b) Mass Spectrum of peak 7 (c) Mass Spectrum of peak 22 (d) Mass Spectrum of peak 23; Figure S6. Mass Spectra of biologically active compounds of fraction K of *Ganoderma lucidum*. (a) Mass Spectrum of peak 7 (b) Mass Spectrum of peak 11 (c) Mass Spectrum of peak 19 (d) Mass Spectrum of peak 26 (e) Mass Spectrum of peak 28 (f) Mass Spectrum of peak 31; Figure S7. Mass Spectra of biologically active compounds of fraction L of Ganoderma lucidum. (a) Mass Spectrum of peak 1 (b) Mass Spectrum of peak 11 (c) Mass Spectrum of peak 16 (d) Mass Spectrum of peak 22; Figure S8. Mass Spectra of biological active compounds of fraction M of *Ganoderma lucidum*. (a) Mass Spectrum of peak 8 (b) Mass Spectrum of peak 10 (c) Mass Spectrum of peak 24; Figure S9. Mass Spectra of biologically active compounds of fraction N of *Ganoderma lucidum*. (a) Mass Spectrum of peak 5 (b) Mass Spectrum of peak 10 (c) Mass Spectrum of peak 14 (d) Mass Spectrum of peak 16 (e) Mass Spectrum of peak 26; Figure S10. Mass Spectra of biologically active compounds of fraction O of *Ganoderma lucidum*. (a) Mass Spectrum of peak 5 (b) Mass Spectrum of peak 15 (c) Mass Spectrum of peak 17 (d) Mass Spectrum of peak 30; Figure S11. Mass Spectra of biologically active compounds of fraction P of *Ganoderma lucidum*. (a) Mass Spectrum of peak 4 (b) Mass Spectrum of peak 6; Table S1. One-Way ANOVA Summary for Antioxidant Activity (DPPH Assay); Table S2. One-Way ANOVA Summary for α-Amylase Inhibition; Table S3. One-Way ANOVA Summary for α-Glucosidase Inhibition; Table S4. Two-Way ANOVA Summary for Antimicrobial Activity; Table S5. Summary of Statistical Tests Used.
