# Supplementary material for: Phytochemical Profiling and Bioactivity Evaluation of Ganoderma lucidum (Reishi Mushroom) Fractions: In Vitro Antioxidant, Antimicrobial, and Antidiabetic Activities
Source: Metabolites. 2026 Mar 30;16(4):225. doi: 10.3390/metabo16040225 (PMC13117725; doi:10.3390/metabo16040225)
Supplement: Supplementary file 1 [file metabolites-16-00225-s001.zip › metabolites-4153390-supplementary.pdf]

metabolites

MDPI

Supporting information

Phytochemical Profiling and Bioactivity Evaluation of *Ganoderma lucidum* (Reishi Mushroom) Fractions: In Vitro Antioxidant, Antimicrobial, and Antidiabetic Activities

Neelum Shehzadi[1], Muhammad Afzal[1,], Sarmir Khan[2], Leonardo Degennaro[2], Gabriele D'Arienzo[3], Noshaba Mehmood[1], Aqsa Chaudhary[1] and Maria Pia Argentieri [2*]

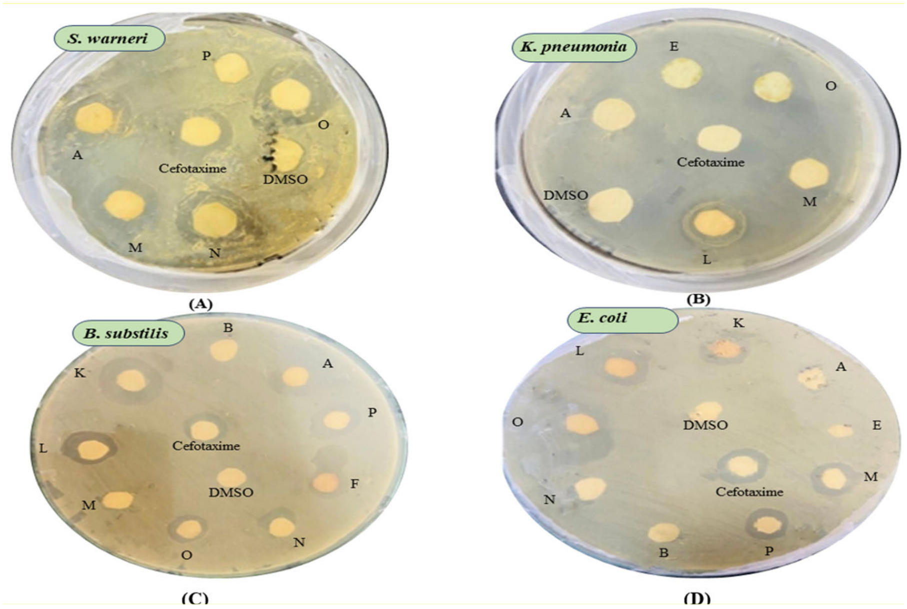

Figure S1. Inhibition zones in mm (a) *S. warneri,* (b) *K. pneumoniae,* (c) *B. substilis,* (d) *E. coli*

 https://doi.org/10.3390/metabo16040225

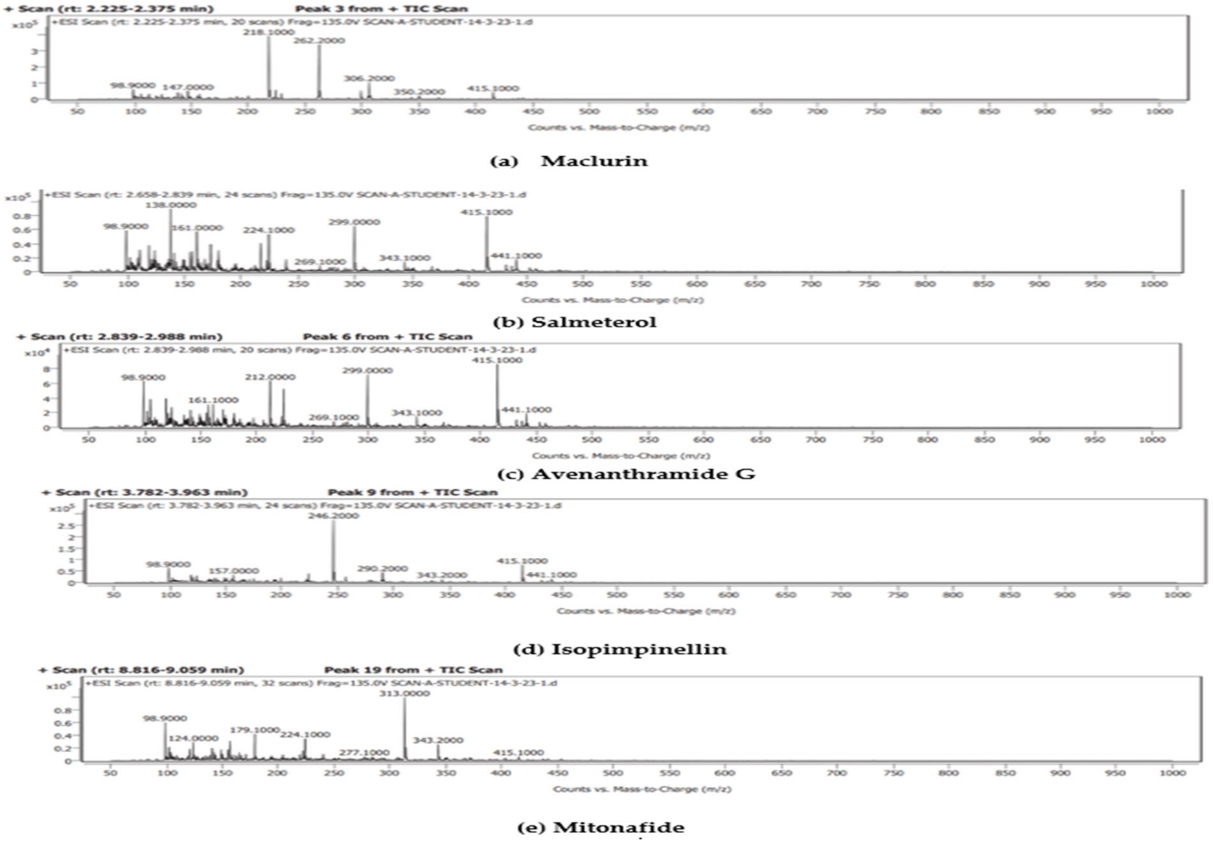

Figure S2. Mass Spectra of biologically active compounds of fraction A of *Ganoderma lucidum*. (**a**) Mass Spectrum of peak 3 (**b**) Mass Spectrum of peak 5 (**c**) Mass Spectrum of peak 6 (**d**) Mass Spectrum of peak 9 (**e**) Mass Spectrum of peak 19

https://doi.org/10.3390/metabo16040225

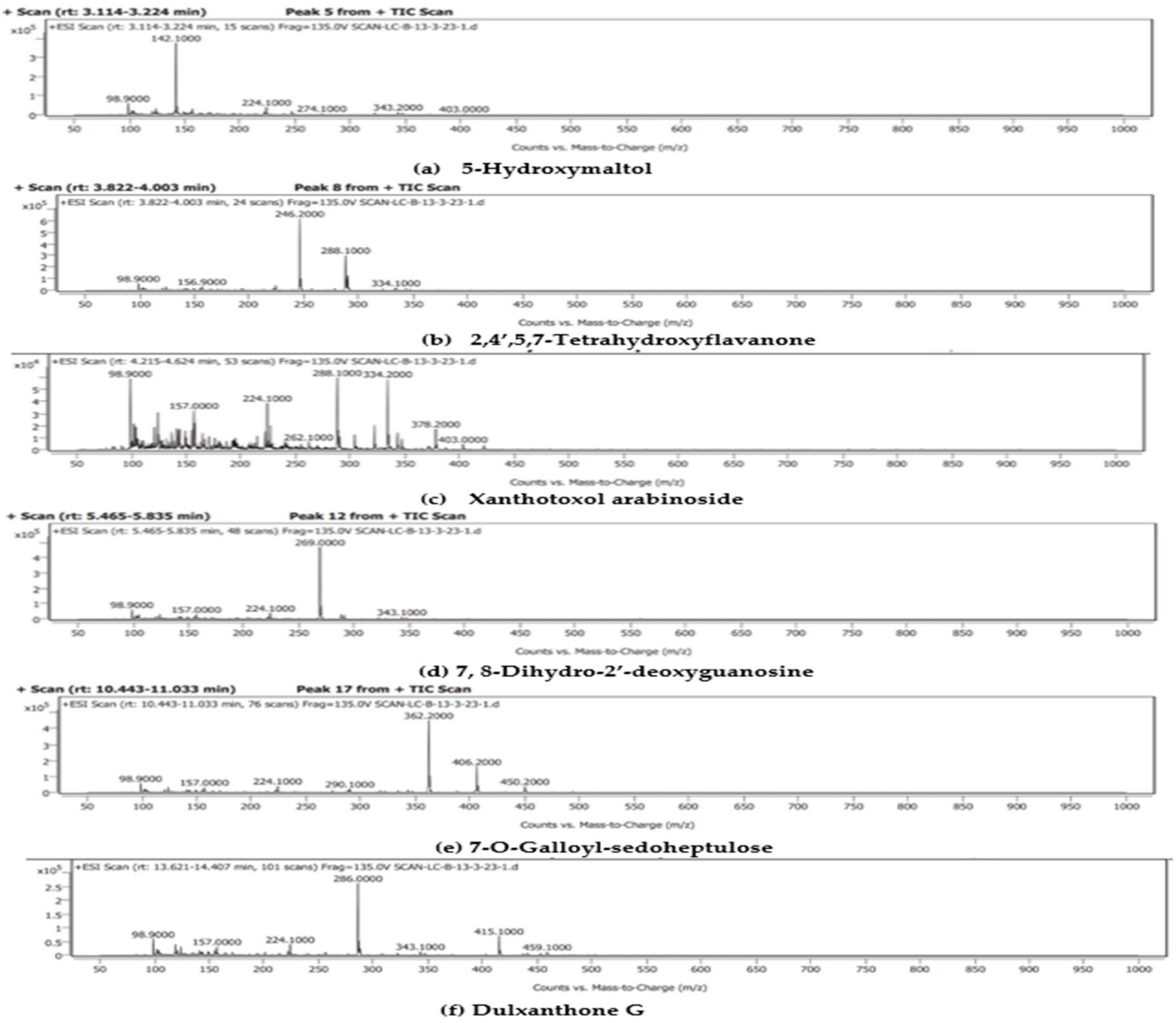

Figure S3. Mass Spectra of biologically active compounds of fraction B of *Ganoderma lucidum*. (a) Mass Spectrum of peak 5 (b) Mass Spectrum of peak 8 (c) Mass Spectrum of peak 10 (d) Mass Spectrum of peak 12 (e) Mass Spectrum of peak 17 (f) Mass Spectrum of peak 20

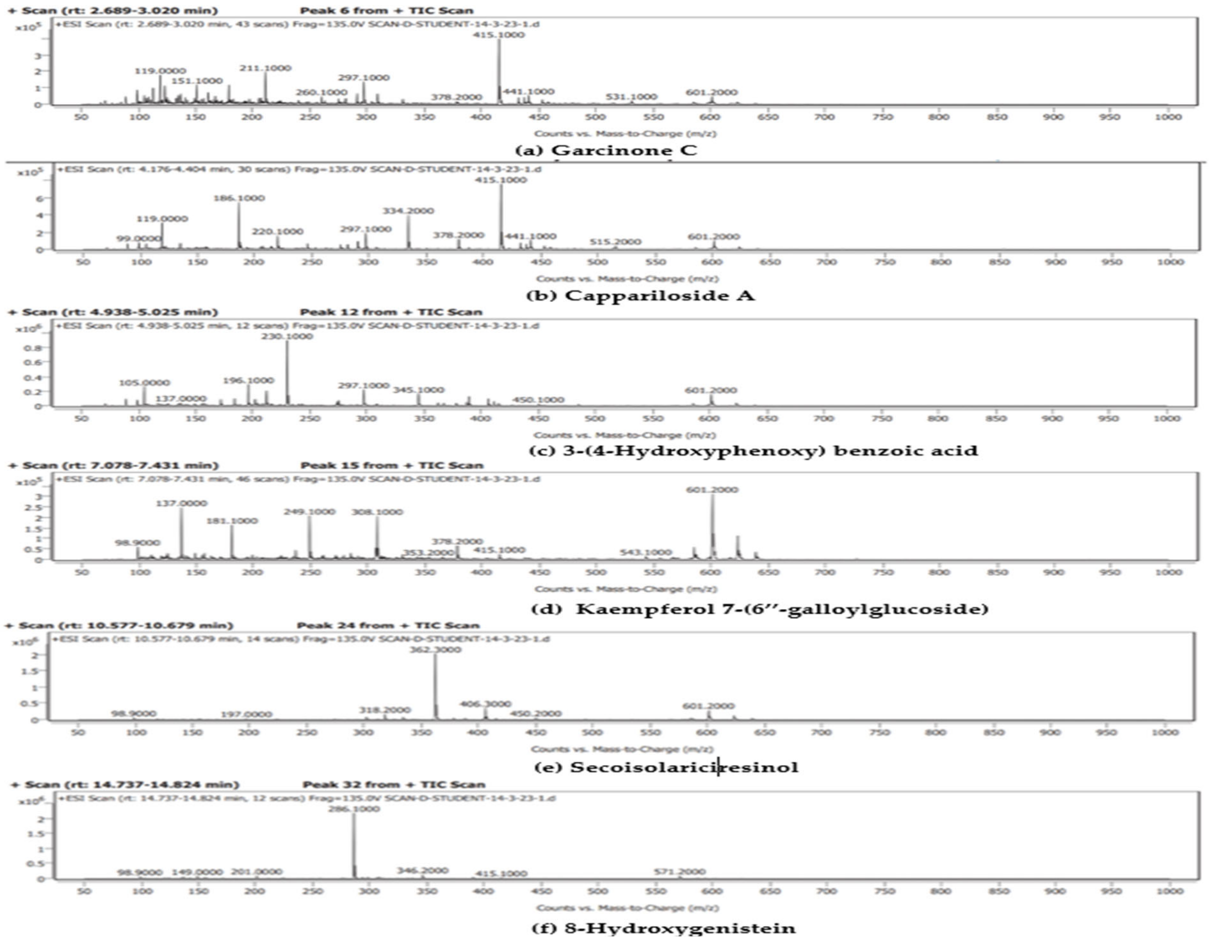

Figure S4 Mass Spectra of biologically active compounds of fraction E of *Ganoderma lucidum*. (a) Mass Spectrum of peak 6 (b) Mass Spectrum of peak 10 (c) Mass Spectrum of peak 12 (d) Mass Spectrum of peak 15 (e) Mass Spectrum of peak 24 (f) Mass Spectrum of peak 32

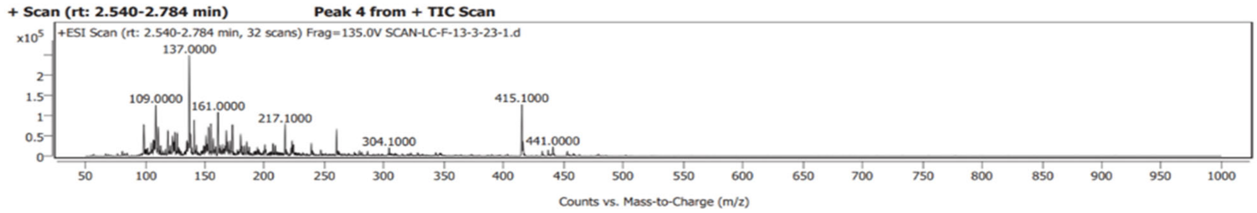

(a) 2,6-Dihydroxy-3-cyanopyridine

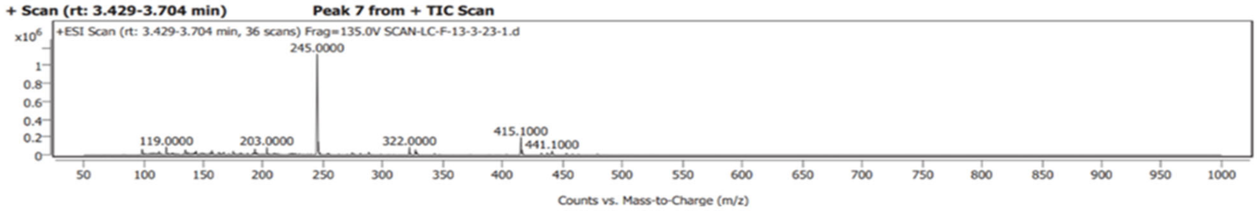

(b) Eriobofuran

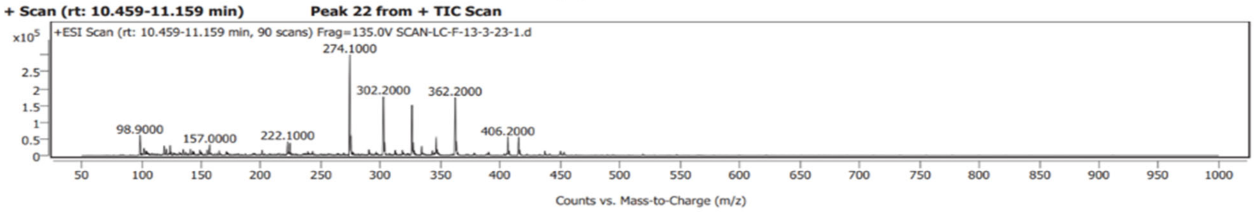

(c) 1,3,6-Trihydroxy-5-methoxyxanthone

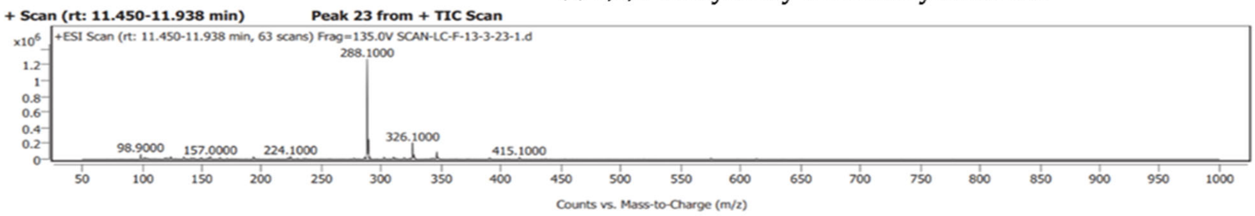

(d) Eriodictyol

Figure S5. Mass Spectrum of biologically active compounds of fraction F of *Ganoderma lucidum*. (a) Mass Spectrum of peak 4 (b) Mass Spectrum of peak 7 (c) Mass Spectrum of peak 22 (d) Mass Spectrum of peak 23

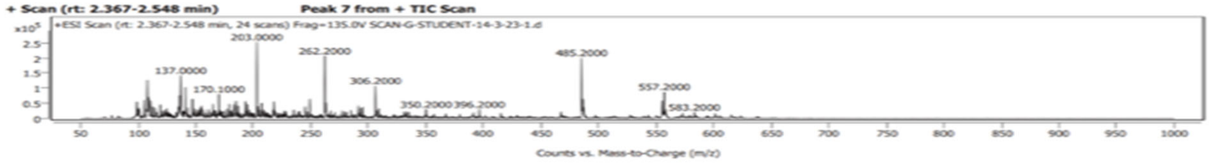

(a) Gallic acid 3-O-(6-galloylglucoside)

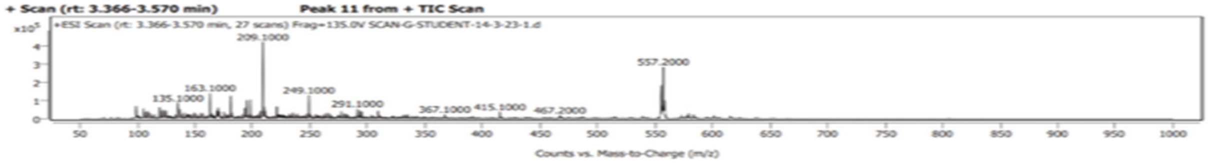

(b) Salvianolic acid K

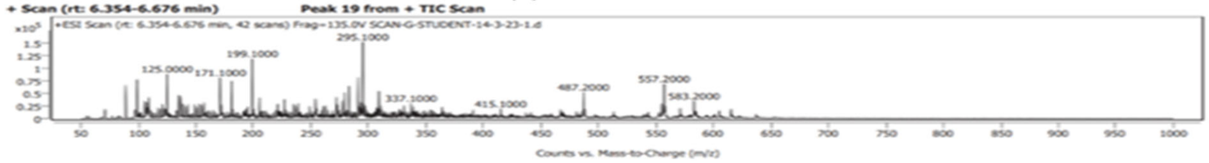

(c) 7-Acetoxy-2-methylisoflavone

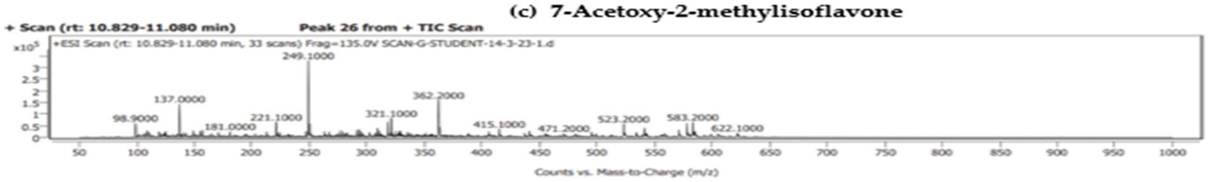

(d) (E)-2-Methyl-2-buten-1-ol O-beta-D-Glucopyranoside

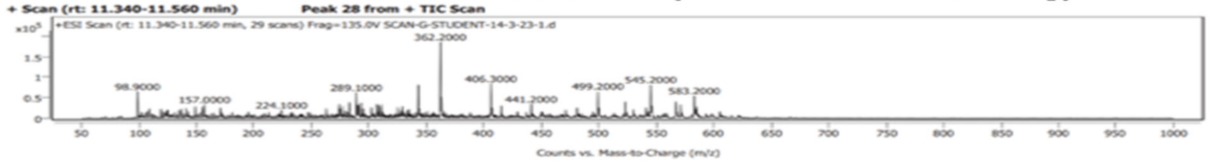

(e) Avenanthramide A2

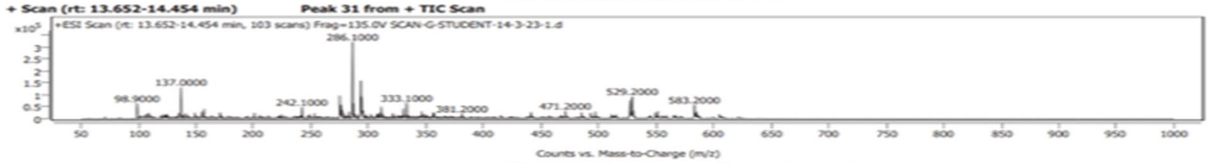

(f) 5,7-Dihydroxy-4'-methoxyflavanone

Figure S6. Mass Spectra of biologically active compounds of fraction K of *Ganoderma lucidum*. (a) Mass Spectrum of peak 7 (b) Mass Spectrum of peak 11 (c) Mass Spectrum of peak 19 (d) Mass Spectrum of peak 26 (e) Mass Spectrum of peak 28 (f) Mass Spectrum of peak 31

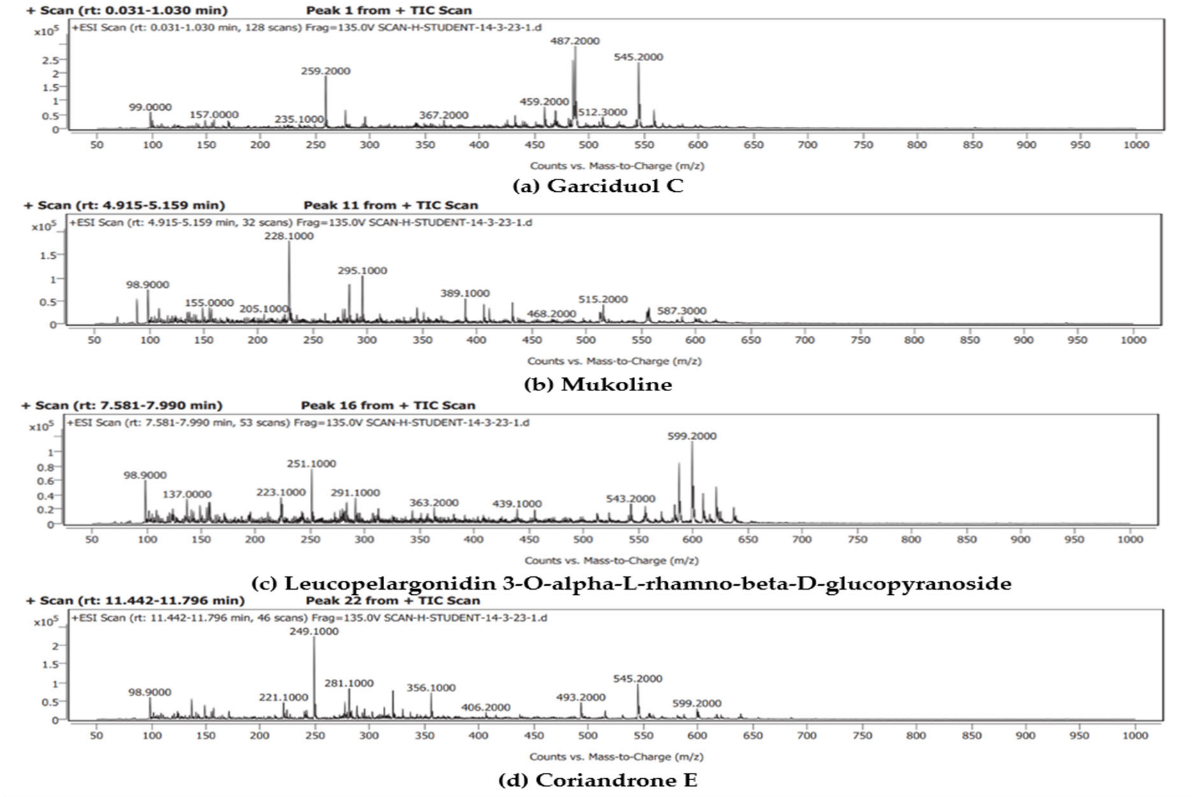

Figure S7. Mass Spectra of biologically active compounds of fraction L of Ganoderma lucidum. (a) Mass Spectrum of peak 1 (b) Mass Spectrum of peak 11 (c) Mass Spectrum of peak 16 (d) Mass Spectrum of peak 22

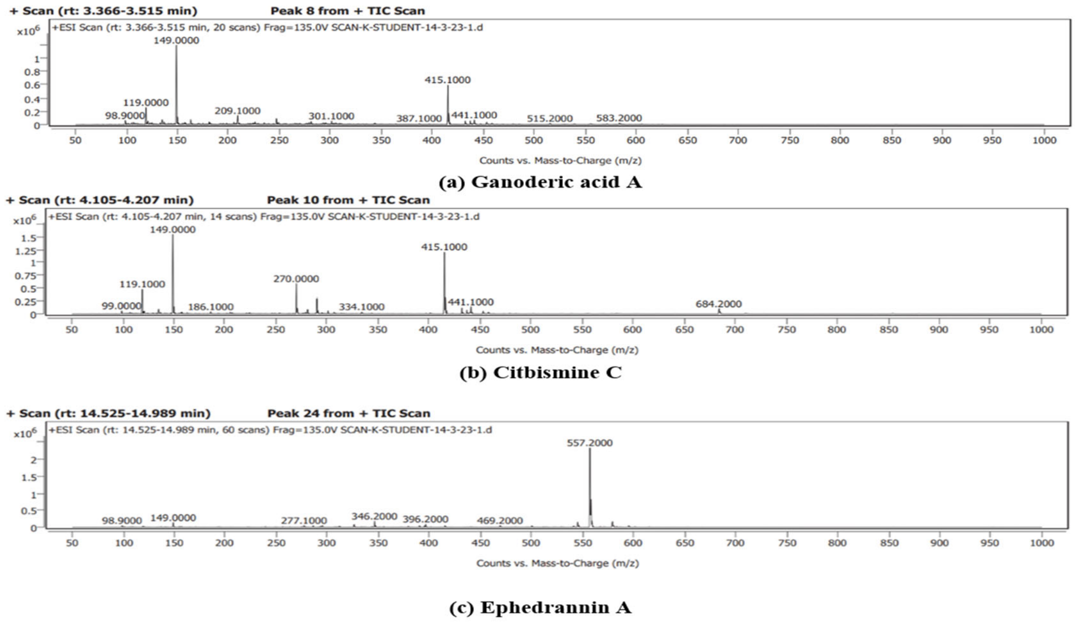

Figure S8. Mass Spectra of biological active compounds of fraction M of *Ganoderma lucidum*. (a) Mass Spectrum of peak 8 (b) Mass Spectrum of peak 10 (c) Mass Spectrum of peak 24

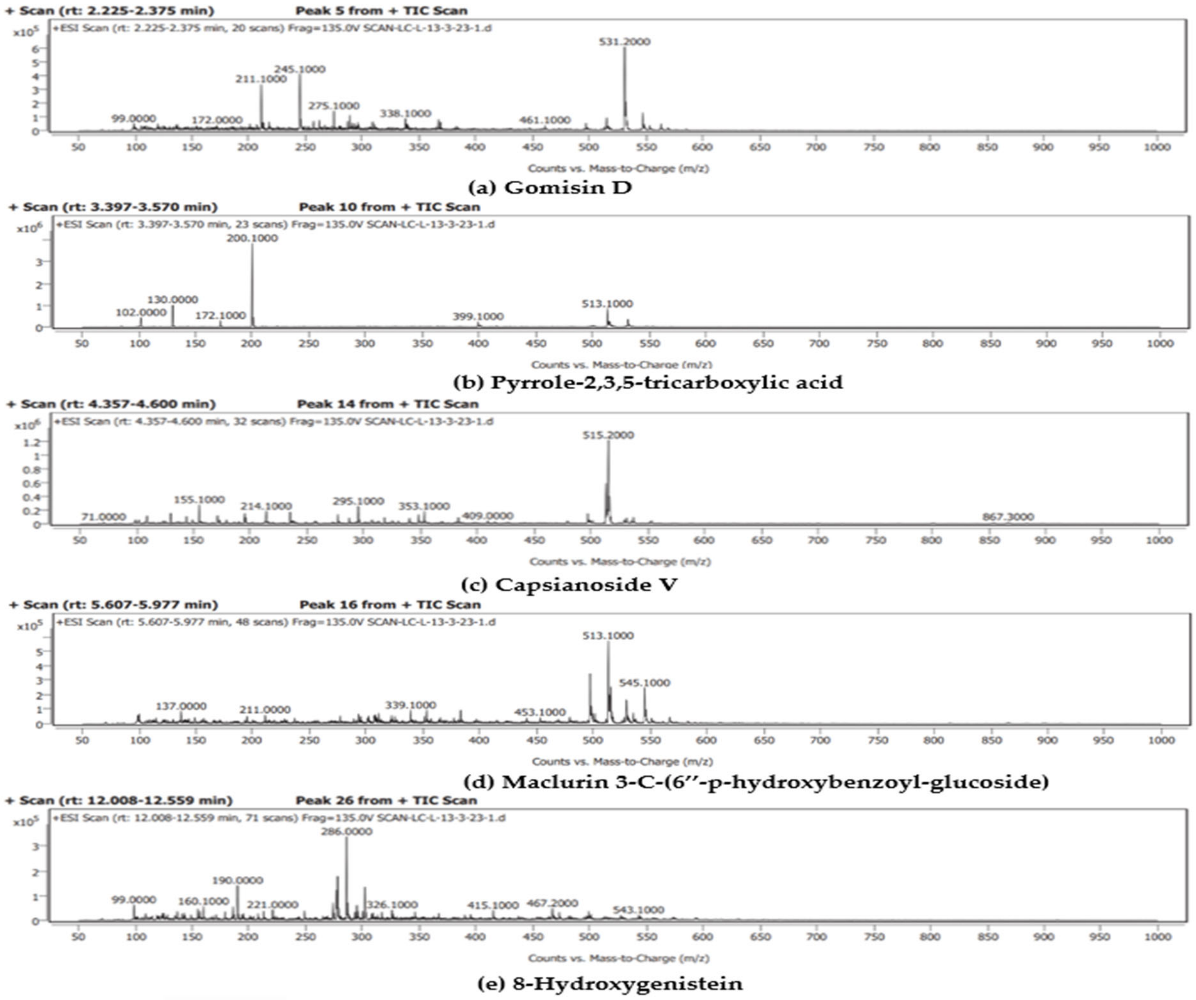

Figure S9. Mass Spectra of biologically active compounds of fraction N of *Ganoderma lucidum*. (a) Mass Spectrum of peak 5 (b) Mass Spectrum of peak 10 (c) Mass Spectrum of peak 14 (d) Mass Spectrum of peak 16 (e) Mass Spectrum of peak 26

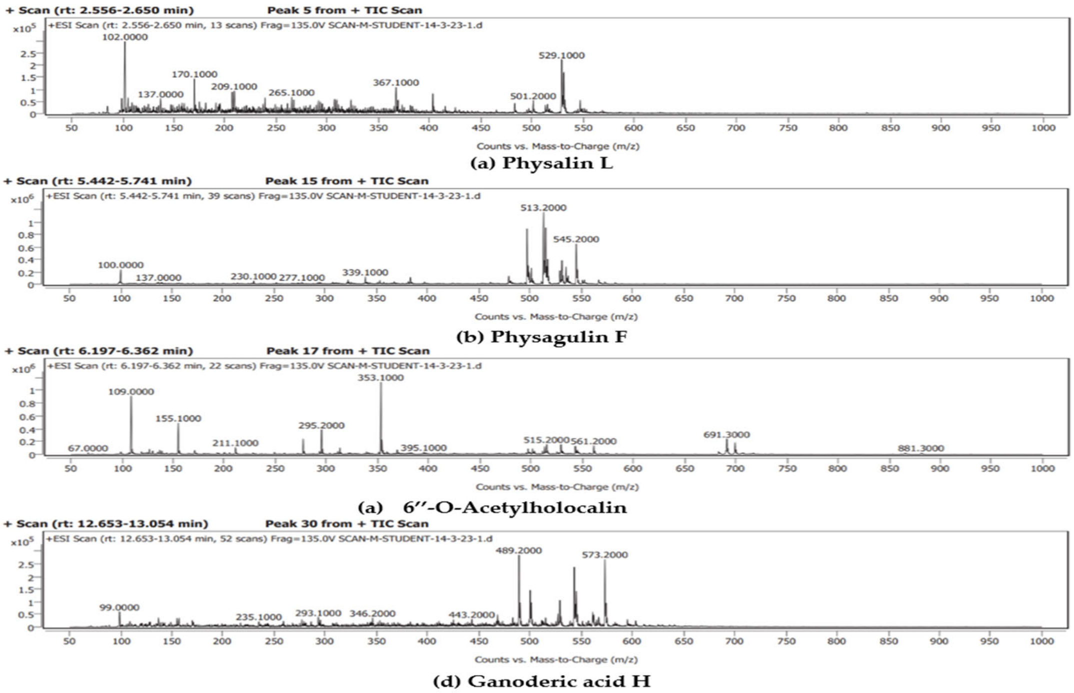

Figure S10. Mass Spectra of biologically active compounds of fraction O of *Ganoderma lucidum*. (a) Mass Spectrum of peak 5 (b) Mass Spectrum of peak 15 (c) Mass Spectrum of peak 17 (d) Mass Spectrum of peak 30

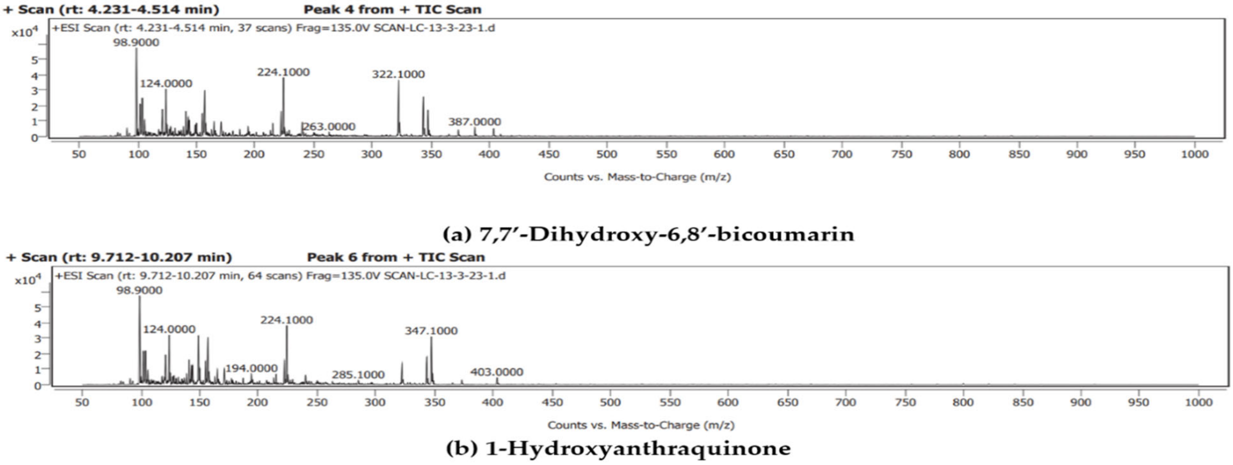

Figure S11. Mass Spectra of biologically active compounds of fraction P of *Ganoderma lucidum*. (a) Mass Spectrum of peak 4 (b) Mass Spectrum of peak 6

Table S1. One-Way ANOVA Summary for Antioxidant Activity (DPPH Assay)

**Source of Variation**	**Sum of Squares (SS)**	**Degrees of Freedom (df)**	**Mean Square (MS)**	**F-value**	**p-value**
Between Groups	12.847	10	1.285	28.47	< 0.0001
Within Groups	0.994	22	0.045		
**Total**	**13.841**	**32**			

https://doi.org/10.3390/metabo16040225

Table: Post-hoc: Dunnett's Multiple Comparisons Test vs. Ascorbic Acid

**Comparison**	**Mean Difference**	**95% CI of Diff.**	**Adjusted p-value**
A vs. Ascorbic	0.57	0.12 to 1.02	0.008
B vs. Ascorbic	0.50	0.05 to 0.95	0.024
E vs. Ascorbic	2.55	2.10 to 3.00	< 0.0001
F vs. Ascorbic	0.55	0.10 to 1.00	0.012
K vs. Ascorbic	0.42	-0.03 to 0.87	0.082
L vs. Ascorbic	0.13	-0.32 to 0.58	> 0.999
M vs. Ascorbic	0.93	0.48 to 1.38	< 0.0001
N vs. Ascorbic	2.17	1.72 to 2.62	< 0.0001
O vs. Ascorbic	2.35	1.90 to 2.80	< 0.0001
P vs. Ascorbic	1.13	0.68 to 1.58	< 0.0001

Normality Tests: Shapiro-Wilk W = 0.94, p = 0.52; Skewness = -0.42 to 0.76; Kurtosis = -0.89 to 1.24

Table S2. One-Way ANOVA Summary for α-Amylase Inhibition

**Source of Variation**	**Sum of Squares (SS)**	**Degrees of Freedom (df)**	**Mean Square (MS)**	**F-value**	**p-value**
Between Groups	15.234	10	1.523	34.82	< 0.0001
Within Groups	0.962	22	0.044		
**Total**	**16.196**	**32**			

Post-hoc: Dunnett's Multiple Comparisons Test vs. Metformin

**Comparison**	**Mean Difference**	**95% CI of Diff.**	**Adjusted p-value**
A vs. Metformin	0.67	0.22 to 1.12	0.002

Comparison	Mean Difference	95% CI of Diff.	Adjusted p-value
B vs. Metformin	0.05	-0.40 to 0.50	> 0.999
E vs. Metformin	2.09	1.64 to 2.54	< 0.0001
F vs. Metformin	0.27	-0.18 to 0.72	0.412
K vs. Metformin	0.59	0.14 to 1.04	0.006
L vs. Metformin	0.28	-0.17 to 0.73	0.358
M vs. Metformin	0.19	-0.26 to 0.64	0.721
N vs. Metformin	0.50	0.05 to 0.95	0.028
O vs. Metformin	0.89	0.44 to 1.34	< 0.0001
P vs. Metformin	2.95	2.50 to 3.40	< 0.0001

Normality Tests: Shapiro-Wilk W = 0.96, p = 0.38; Skewness = -0.65 to 0.82; Kurtosis = -1.12 to 0.95

Table S3. One-Way ANOVA Summary for α-Glucosidase Inhibition

Source of Variation	Sum of Squares (SS)	Degrees of Freedom (df)	Mean Square (MS)	F-value	p-value
Between Groups	18.456	10	1.846	42.15	< 0.0001
Within Groups	0.964	22	0.044		
**Total**	**19.420**	**32**			

Post-hoc: Dunnett's Multiple Comparisons Test vs. Metformin

Comparison	Mean Difference	95% CI of Diff.	Adjusted p-value
A vs. Metformin	3.18	2.73 to 3.63	< 0.0001
B vs. Metformin	1.90	1.45 to 2.35	< 0.0001
E vs. Metformin	0.08	-0.37 to 0.53	> 0.999

Comparison	Mean Difference	95% CI of Diff.	Adjusted p-value
F vs. Metformin	1.62	1.17 to 2.07	< 0.0001
K vs. Metformin	0.73	0.28 to 1.18	0.002
L vs. Metformin	0.20	-0.25 to 0.65	0.682
M vs. Metformin	3.27	2.82 to 3.72	< 0.0001
N vs. Metformin	0.66	0.21 to 1.11	0.005
O vs. Metformin	0.33	-0.12 to 0.78	0.221
P vs. Metformin	2.07	1.62 to 2.52	< 0.0001

Normality Tests: Shapiro-Wilk W = 0.95, p = 0.41; Skewness = -0.58 to 0.91; Kurtosis = -1.05 to 1.18

Table S4. Two-Way ANOVA Summary for Antimicrobial Activity

Source of Variation	Sum of Squares (SS)	df	Mean Square (MS)	F-value	p-value
Fraction	1,247.35	9	138.59	45.23	< 0.0001
Bacterial Strain	89.42	3	29.81	9.73	< 0.0001
Fraction × Strain	456.78	27	16.92	5.52	< 0.0001
Residual	245.12	80	3.06		
**Total**	**2,038.67**	**119**			

Post-hoc: Tukey's HSD Test for Fraction O vs. Others (Pooled Across Bacteria)

Comparison	Mean Difference (mm)	95% CI of Diff.	Adjusted p-value
O vs. A	14.2	10.8 to 17.6	< 0.0001
O vs. B	16.8	13.4 to 20.2	< 0.0001
O vs. E	13.5	10.1 to 16.9	< 0.0001

Comparison	Mean Difference (mm)	95% CI of Diff.	Adjusted p-value
O vs. F	8.3	4.9 to 11.7	< 0.0001
O vs. K	6.8	3.4 to 10.2	0.0002
O vs. L	5.2	1.8 to 8.6	0.0018
O vs. M	11.4	8.0 to 14.8	< 0.0001
O vs. N	5.8	2.4 to 9.2	0.0008
O vs. P	7.5	4.1 to 10.9	< 0.0001

Table S5. Summary of Statistical Tests Used

Assay	Experimental Design	Statistical Test	Post-hoc Test	Key Finding
DPPH (Antioxidant)	10 fractions vs. 1 standard	One-way ANOVA	Dunnett's	Fractions L, B, K ≈ Ascorbic acid ($p > 0.05$)
α-Amylase Inhibition	10 fractions vs. 1 standard	One-way ANOVA	Dunnett's	Fractions B, F, L, M ≈ Metformin ($p > 0.05$)
α-Glucosidase Inhibition	10 fractions vs. 1 standard	One-way ANOVA	Dunnett's	Fractions E, L, O ≈ Metformin ($p > 0.05$)
Antimicrobial	10 fractions × 4 bacterial strains	Two-way ANOVA	Tukey's HSD	Fraction O > all others vs. Gram-negatives ($p < 0.001$)

Normality Assessment: Shapiro-Wilk test; Skewness and kurtosis within ±2.0; Homogeneity of variance: Levene's test ($p > 0.05$ for all analyses). Significance level: $p \le 0.05$. Software: GraphPad Prism 9.0 and SPSS v. 28.

https://doi.org/10.3390/metabo16040225
